# HSPCs and T_reg_ cells cooperate to preserve extramedullary hematopoiesis under chronic inflammation

**DOI:** 10.1126/sciadv.adv9351

**Published:** 2026-05-06

**Authors:** Maria Kuzmina, Srdjan Grusanovic, Jiri Brezina, Flavian Thelen, Karolina Vanickova, Mirko Milosevic, Irina Ribeiro Bas, Nataliia Pavliuchenko, Sarka Ruzickova, Jakub Rohlena, Dominik Filipp, Katerina Rohlenova, Cesar Nombela-Arrieta, Tomas Brdicka, Meritxell Alberich-Jorda

**Affiliations:** ^1^ Laboratory of Hemato-oncology, Institute of Molecular Genetics of the Czech Academy of Sciences, Videnska 1083, 142 20, Prague 4, Prague, Czech Republic.; ^2^ Faculty of Science, Charles University, 128 00 Prague, Czech Republic.; ^3^ Laboratory of Immunobiology, Institute of Molecular Genetics of the Czech Academy of Sciences, Videnska 1083, 142 20, Prague 4, Prague, Czech Republic.; ^4^ Department of Medical Oncology and Hematology, University of Zurich and University Hospital Zurich, Zurich, Switzerland.; ^5^ Laboratory of Cellular Metabolism, Institute of Biotechnology of the Czech Academy of Sciences, Prumyslova 595, 252 50 Vestec, Prague-West, Czech Republic.; ^6^ Laboratory of Leukocyte Signaling, Institute of Molecular Genetics of the Czech Academy of Sciences, Videnska 1083, 142 20 Prague 4, Prague, Czech Republic.; ^7^ Laboratory of Developmental Epileptology, Institute of Physiology of the Czech Academy of Sciences, Videnska 1083, 142 20 Prague 4, Prague, Czech Republic.; ^8^ Childhood Leukaemia Investigation Prague, Department of Pediatric Haematology and Oncology, Second Faculty of Medicine, Charles University in Prague, University Hospital Motol, V Uvalu 84, 150 06 Prague, Czech Republic.

## Abstract

Hematopoietic stem and progenitor cells (HSPCs) are localized within specialized niches of the bone marrow (BM). However, during hematological disorders or infections, the functionality of these cells in the BM is compromised, leading to extramedullary hematopoiesis (EMH). Chronic inflammation drives EMH, yet its impact on HSPCs outside the BM is poorly understood. Using a mouse model of chronic autoinflammatory disease, we demonstrated the presence of extramedullary HSPCs in blood, spleen, and inflamed tails and paws. Single-cell transcriptomics revealed a unique expression profile in extramedullary HSCs, with significant up-regulation of *Cd53*, MHCII-associated, and immunosuppressive genes. We further demonstrated that extramedullary CD53^+^ HSPCs act as antigen-presenting cells, promoting the development of regulatory T cells (T_reg_ cells) to control chronic inflammation at extramedullary sites. Conversely, T_reg_ cells exert a protective role on extramedullary HSPCs. Together, our findings revealed a mutually supportive relationship between a unique subset of HSPCs and T cells in inflamed tissues during chronic inflammation.

## Teaser

HSPCs and T_reg_ cells located at extramedullary sites establish a mutually beneficial relationship during chronic inflammation.

## INTRODUCTION

Hematopoietic stem cells (HSCs) are a rare population of cells characterized by their multipotency, self-renewal capabilities, and low metabolic and proliferative states. Residing within specialized niches in the bone marrow (BM), HSCs are essential for the continuous production of mature blood cells. To prevent excessive cycling and the accumulation of mutations, HSCs remain quiescent (*1*, *2*). Therefore, daily hematopoiesis is predominantly driven by hematopoietic progenitor cells, with minimal contribution from HSCs (*3*, *4*). Under stress conditions, however, HSCs respond to increased hematopoietic demands by enhancing the production of downstream progenitors (*5*, *6*). Extensive research has explored the biology of HSCs within the BM, particularly examining the effects of cell intrinsic and extrinsic factors on their behavior. Nevertheless, the properties of HSCs outside the BM, especially under long-term stress conditions, remain less understood.

Chronic inflammation is a pathological condition characterized by persistent low-grade inflammation with elevated levels of proinflammatory cytokines and chemokines. Numerous studies have demonstrated that such inflammation can compromise HSC function in the BM by inducing replicative stress, leading to DNA damage and stem cell exhaustion (*7*–*9*). The consequences of chronic inflammation extend beyond diminished stem cell functionality, potentially triggering hematopoietic stem and progenitor cell (HSPC) mobilization and migration to peripheral sites (*10*, *11*). This adaptive mechanism, known as extramedullary hematopoiesis (EMH), occurs as hematopoietic cells relocate to organs previously active in fetal hematopoiesis to sustain blood cell production when BM hematopoiesis becomes insufficient. Spleen and liver are notably most well-documented sites for EMH, featuring unique microenvironments distinct from those of the BM (*12*). EMH is often associated with a myeloid-biased output, a hallmark particularly evident in the context of chronic inflammation and cancer (*13*, *14*). Proinflammatory cytokines such as interleukin-1β (IL-1β) and IL-6, which are usually elevated in pathological and stress conditions, induce proliferation and myeloid differentiation of BM HSPCs (*6*, *15*). However, the impact of chronic inflammation on HSPCs located at the sites of EMH remains poorly understood.

To investigate the effect of chronic inflammation on HSPCs located at EMH sites, we used a mouse model of chronic multifocal osteomyelitis (CMO), which resembles human autoinflammatory disease chronic recurrent multifocal osteomyelitis (CRMO) (*16*). CMO mice carry a missense mutation, L98P, in the gene *Pstpip2* coding for proline-serine-threonine phosphatase-interacting protein 2 (Pstpip2). The mutation results in misfolding and consequent degradation of the Pstpip2 protein (*17*, *18*). Pstpip2 is expressed in immune cells, such as macrophages and neutrophils, but it is undetected in HSCs (*18*–*20*). Pstpip2 is known to play an anti-inflammatory role by regulating reactive oxygen species production and cytokine levels, including IL-1β and IL-6 (*18*, *19*, *21*). Consequently, CMO mice exhibit a progressive sterile autoinflammatory disorder, with mice being born healthy and developing disease signs, such as tail kinks and inflamed hind paws, at around 7 weeks of age (*22*). Recently, we demonstrated that IL-6 drives an expansion of the HSC pool and diminishes HSC function in the BM, compromising BM hematopoiesis (*20*).

Here, we demonstrate that extramedullary HSPCs are localized not only in the blood and spleen of CMO mice but also at sites of inflammation such as the paw. Using single-cell transcriptomics, we identified *Cd53* as a top differentially expressed gene in extramedullary HSPCs. This observation was coupled with the up-regulation of major histocompatibility complex class II (MHCII)–associated and immunosuppressive genes. Moreover, we showed that extramedullary CD53^+^ HSPCs function as antigen-presenting cells (APCs), facilitating the development of regulatory T cells (T_reg_ cells) that potentially mitigate chronic inflammation at extramedullary hematopoietic sites. Additionally, we observed that T_reg_ cells play a protective role for extramedullary HSPCs. Together, our findings unveil a bidirectional interaction between a distinct subset of HSPCs and T cells in inflamed tissues during chronic inflammation.

## RESULTS

### CMO mice exhibit increased numbers of functional HSPCs in PB and spleen

**Fig. 1.**
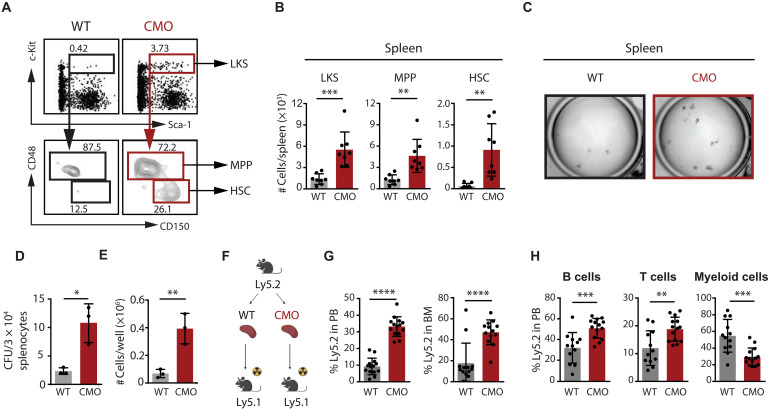
CMO mice show increased levels of functional HSPCs in spleen. (**A**) Representative flow cytometry plots from 1 WT and 1 CMO spleen. Top plots indicate c-Kit and Sca-1 levels in lineage^−^ (Lin^−^) splenocytes. Gates indicate Lin^−^ c-Kit^+^ Sca-1^+^ (LKS) cells. Bottom plots show CD48 and CD150 expression in LKS, and gates indicate LKS CD48^+^ CD150^−^ cells (MPPs) and LKS CD48^−^ CD150^+^ cells (HSCs). Numbers show percentage from parental gates. (**B**) Quantification of (A). The *y* axes indicate the number of LKS cells, MPPs, and HSCs in WT and CMO spleens. Eight to 10 mice were included per group in three independent experiments. Each mouse is represented by a dot symbol. (**C**) Representative microscopy images of colony culture assays at day 7 of culture. WT and CMO splenocytes (3 × 10^4^) were plated per well. (**D** and **E**) Number of colony-forming units (CFU) (D) and cells (E) enumerated in CFU assays at day 7. The *y* axes indicate numbers per well. At least three spleens were used in each condition. (**F**) Schematic representation of the transplantation setup. Created in BioRender. Alberich jorda, M. (2026) https://BioRender.com/2qxjbs1 (**G**) Engraftment of WT or CMO splenocytes upon transplantation into lethally irradiated congenic recipients. The *y* axis indicates percentage of donor-derived Ly5.2^+^ cells in peripheral blood (PB; left) and BM (right) of recipient mice 16 weeks posttransplantation. Twelve recipients were used per group in two independent experiments. Each dot symbol indicates values per one recipient. (**H**) Trilineage reconstitution analysis in PB of recipient mice 16 weeks after transplantation. The *y* axis indicates the percentage of donor-derived Ly5.2^+^ B, T, and myeloid cells. Each dot indicates values for one recipient mouse, and at least 12 mice were used in each group. In this figure, 12- to 25-week-old male and female mice were used. Data indicate means ± SD, and two-tailed Student’s *t* test was used to assess statistical significance (*P < 0.05; **P < 0.01; *** *P* < 0.001; ****P < 0.0001).

Because hematopoiesis can occur outside of the BM under inflammatory stress (*23*–*25*), we explored whether our mouse model of sterile chronic inflammation exhibits EMH. We assessed the presence of HSPCs in peripheral blood (PB) and spleen of wild-type (WT) and CMO mice and observed an increased number of Lin^−^ c-Kit^+^ Sca-1^+^ (LKS), multipotent progenitors (MPPs; LKS CD48^+^ CD150^−^), and HSCs (LKS CD48^−^ CD150^+^) in CMO samples in comparison with WT (Fig. 1, A and B; and fig. S1, A to C). These circulating and spleen-resident HSPCs were functional, as evidenced by a significant increase in colony numbers observed in cultures established from CMO as opposed to WT cells (Fig. 1, C to E; and fig. S1, D to F). Next, we assessed the functionality of extramedullary HSPCs in vivo by transplanting blood and splenocytes isolated from WT or CMO mice (Ly5.2) into lethally irradiated Ly5.1 congenic recipients (Fig. 1F and fig. S1G). Consistent with our in vitro results, analysis of blood and BM from recipient mice 16 weeks after transplantation revealed increased engraftment in recipients transplanted with CMO blood and splenocytes compared with recipients transplanted with WT control cells (Fig. 1G and fig. S1H). Although CMO mice exhibit an expansion of the myeloid lineage (*18*, *20*), we did not detect a myeloid bias in the blood and spleen of mice transplanted with CMO samples. Instead, we observed enhanced lymphoid production (Fig. 1H and fig. S1, I and J). Together, these experiments indicate that CMO mice display an increased number of phenotypical and functional HSPCs in circulation and spleen, supporting the existence of EMH in CMO mice.

### CMO mice host HSPCs at the sites of inflammation

**Fig. 2.**
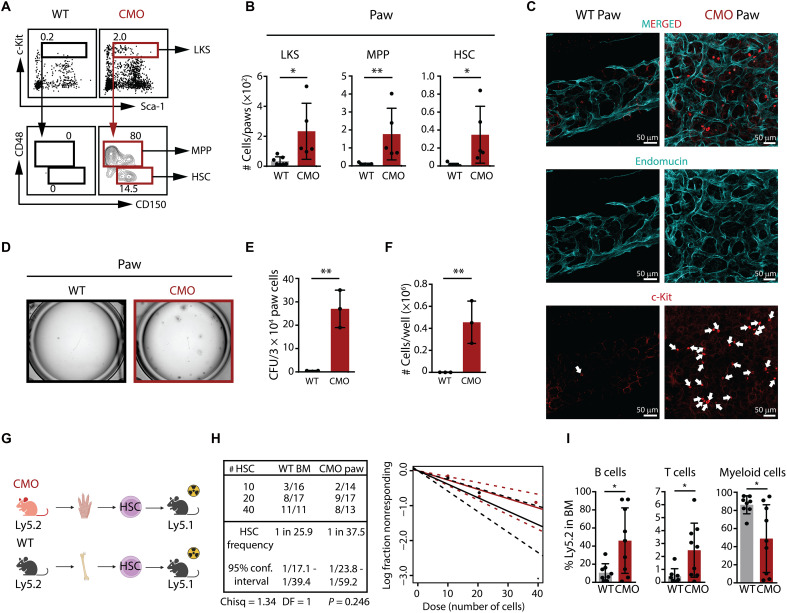
CMO mice exhibit ongoing EMH at inflamed paws. (**A**) Flow cytometry plots from WT and CMO paw cell suspension. Top plots indicate c-Kit and Sca-1 expression in lineage^−^ cells. Bottom plots indicate CD48 and CD150 expression in LKS. The numbers indicate the percentage from parental gates. (**B**) Quantification of (A). The *y* axes indicate number of LKS, MPP, and HSC. At least five mice were included per group in two independent experiments. Each mouse is represented by a dot. (**C**) Representative confocal images of WT (left) and CMO (right) paw showing endomucin (blue) and c-Kit (red). (**D**) Representative microscopy images of colony assays after 7 days of culture. WT and CMO paw cells (3 × 10^4^) were plated. (**E** and **F**) Number of CFU (E) and cells (F) in (D). The *y* axes indicate numbers at day 7. Paw cells from three mice were used in each condition. (**G**) Schematic of transplantation setup. Created in BioRender. Alberich jorda, M. (2026) https://BioRender.com/sua5dmk. (**H**) Frequency of functional WT BM and CMO paw HSCs measured by transplantation and calculated using ELDA software based on Poisson distribution statistics (chi-square test; Chisq = 1.34; *P* = 0.246). Graph shows the curve fit of the log fraction of nonresponding mice (solid lines) and confidence intervals (dashed lines) versus the number of mice tested. The *x* axis indicates dose of transplanted cells, and the *y* axis indicates percentages of nonresponders. A mouse was a responder when engraftment in BM was >0.1%, and at least two lineages were reconstituted by donor HSCs. Data compile three independent experiments. (**I**) Trilineage reconstitution in BM from responders that received 20 WT BM or CMO paw HSCs. The *y* axes indicate percentage of donor-derived B, T, and myeloid cells. Each dot symbolizes one responder. This figure includes 12- to 25-week-old male and female mice. [(B), (E), (F), and (I)] Data indicate means ± SD, and two-tailed Student’s *t* test was used to assess statistical significance (*P < 0.05; **P < 0.01).

Because CMO mice exhibit signs of EMH, we next investigated whether this model of sterile chronic inflammation would harbor HSPCs at the sites of inflammation. Thus, we assessed the presence of HSPCs in tails and/or paws, sites commonly inflamed in CMO mice and characterized by inflammatory bone damage, high cytokine levels, and elevated number of granulocytes (*18*, *21*, *26*). As expected, WT paws were almost completely devoid of HSPCs, whereas, in inflamed CMO paws, HSPCs were readily detected (Fig. 2, A and B, and fig. S2A). Analysis of CMO paired front and hind paws showed that the presence of HSPCs was restricted to the inflamed hind paws, while the noninflamed front paws contained no detectable HSPCs (fig. S2B), suggesting that alternative, nonclassical, EMH can arise in novel sites with ongoing tissue inflammation. In line with our flow cytometry data, high-resolution confocal microscopy revealed that, while c-kit^+^ cells could be found only seldomly in the WT paws, CMO hind paws displayed a marked increase of this population (Fig. 2C). These c-kit^+^ cells in CMO paw were located inside the bone, frequently in close proximity to blood vessels (Fig. 2C and movies S1 and S2). Therefore, we expand the meaning of EMH to include these HSPCs in CMO hind paws as noninflamed paw BM is devoid of in situ hematopoiesis. Next, colony-forming assays demonstrated that cell suspensions from WT paws and tails were not able to form colonies, whereas those from CMO paws and tails produced multiple colonies, indicating the presence of functional HSPCs at the inflammatory sites (Fig. 2, D to F; and fig. S2, C to E). To further delineate the functionality of CMO paw HSCs in vivo, we performed extreme limiting dilution transplantation assays. As depicted in Fig. 2G, CMO HSCs were sorted from paws, mixed with BM support, and transplanted into lethally irradiated congenic recipients. Given the lack of HSCs in WT paws, we compared the engraftment efficacy of CMO paw HSCs with WT BM HSCs. CMO paw HSCs were able to engraft and reconstitute the hematopoietic system of lethally irradiated recipient mice similarly to WT BM HSCs (Fig. 2H and fig. S2, F and G). Further, despite the significant increase of granulocytes in paws from CMO mice, CMO paw HSCs exhibited a reduced contribution to the myeloid lineage and a significant increase of lymphocyte production (Fig. 2I). Further, to separate the contribution of the BM niche versus hematopoietic cells to the generation of EMH, we generated BM chimeras. We transplanted CMO BM cells into lethally irradiated WT recipient mice, which, 16 weeks later, demonstrated that transfer of CMO BM cells was sufficient to reproduce the full CMO phenotype, including splenomegaly, inflammation of tails and paws, and EMH (fig. S2H) (*18*, *27*, *28*). On the contrary, transplantation of WT BM cells into lethally irradiated CMO recipients demonstrated that transfer of WT BM cells into CMO mice abolished the CMO phenotype, including EMH (fig. S2H) (*18*, *27*, *28*). Together, these results indicate that Pstpip2 deficiency within the hematopoietic compartment is both necessary and sufficient to drive chronic inflammation and EMH and demonstrate the presence of functional HSCs able to efficiently execute hematopoiesis within the heavily inflamed tissues characteristic of the CMO mice, with a differentiation preference toward the lymphoid lineage.

### Extramedullary HSCs exhibit a unique transcriptional profile

**Fig. 3.**
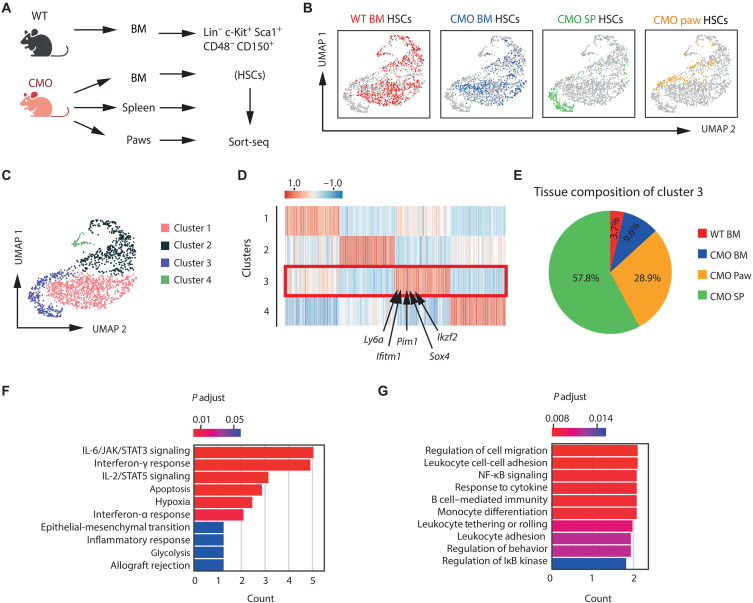
HSCs located in extramedullary sites exhibit a unique transcriptional profile. (**A**) Graphical representation showing the isolation and sorting of HSCs from different origins. HSCs were defined as Lin^−^ c-Kit^+^ Sca-1^+^ CD48^−^ and CD150^+^ cells and sorted from 19-week-old female mice for subsequent Sort-seq analysis. Created in BioRender. Alberich jorda, M. (2026) https://BioRender.com/htysa27. (**B**) UMAP plots color coded for HSCs isolated from the distinct tissues: WT BM HSCs (red), CMO BM HSCs (blue), CMO spleen HSCs (green), and CMO paw HSCs (orange). (**C**) UMAP plot of HSC transcriptomes displaying four clusters identified through unsupervised clustering. (**D**) Relative gene expression levels of 50 top-ranking marker genes for each identified cluster. Red box highlights cluster 3, and several genes significantly up-regulated are indicated. (**E**) Relative contribution of WT BM, CMO BM, CMO spleen, and CMO paw HSCs to the cellular composition of cluster 3. (**F** and **G**) Relevant results of enrichment analysis of differentially expressed genes by Enrichr (*66*) in cluster 3. JAK, Janus kinase; STAT3, signal transducer and activator of transcription 3; NF-κB, nuclear factor κB; I-κB, inhibitor of nuclear factor κB.

To explore potentially distinct transcriptional profiles of HSCs located in different tissues under chronic inflammatory conditions, we used single-cell RNA sequencing (Sort-seq), a technique that enables the transcriptomic analysis of a low number of cells. As illustrated in Fig. 3A, HSCs were sorted from CMO BM, CMO spleen, and CMO paw, as well as from WT BM, and subjected to Sort-seq. The HSC transcriptional profile was notably different in the HSC populations analyzed (Fig. 3B), and unsupervised clustering allowed us to identify four different HSC identities (cluster 1 to cluster 4) (Fig. 3C). This distribution was defined by specific gene expression patterns in each cluster (Fig. 3D and table S1). Cluster 1 gathered HSCs from WT as well as CMO BM, and enrichment analysis of the genes defining this cluster revealed significant enrichment for transcripts associated with stemness (fig. S3, A to E). Further, cluster 1 exhibited significant expression of myelo-inflammatory genes (fig. S3F). We hypothesized that this proinflammatory signature was driven by the CMO BM HSCs, as we previously published that these cells exhibited a myelo-inflammatory identify (*20*). Accordingly, analysis of the differentially expressed genes in WT and CMO BM HSCs demonstrated 1448 down-regulated and 407 up-regulated genes in CMO BM HSCs compared with WT BM HSCs (*P* < 0.05) (fig. S3G), and gene set enrichment analysis corroborated the hyperactivation of the IL-6/Janus kinase/signal transducer and activator of transcription 3 signaling pathway (fig. S3H) as previously reported (*20*). Further, we identified two clusters, clusters 2 and 4, which also compiled HSCs from WT and CMO BM as well as a smaller proportion of extramedullary HSCs (fig. S3A). These clusters were enriched for the presence of transcripts associated with cell cycle, defining a larger subpopulation of HSCs in the G_1_-S phase (cluster 2) and a smaller subpopulation of HSCs in the mitotic phase (cluster 4) (fig. S3, I to L). The analysis revealed that HSCs from the CMO spleen and paw predominantly formed a unique cluster, referred here as cluster 3, with minimal overlap to WT and CMO BM HSCs (Fig. 3, C to E). The identification of cluster 3 indicated that HSCs located at the sites of EMH in CMO mice share a unique transcriptional profile, suggesting the existence of unique biological properties and cellular characteristics (Fig. 3D). Analysis of the top differentially expressed genes in this extramedullary cluster revealed enrichment of transcripts associated with cytokine signaling and adhesion/migration (Fig. 3, F and G). Further, we assessed the expression of specific gene signatures and observed that cluster 3 had a strong lineage bias and inflammatory identity while exhibiting reduced stemness, in comparison with the other clusters (fig. S3, D to F). Together, we observed that HSCs isolated from extramedullary sites in CMO mice share a unique transcriptional profile distinct from BM HSCs, suggesting that inflammation in the extramedullary sites defines the transcriptional properties of these HSCs.

### CD53 expression is increased in extramedullary HSPCs characterized by low proliferation

**Fig. 4.**
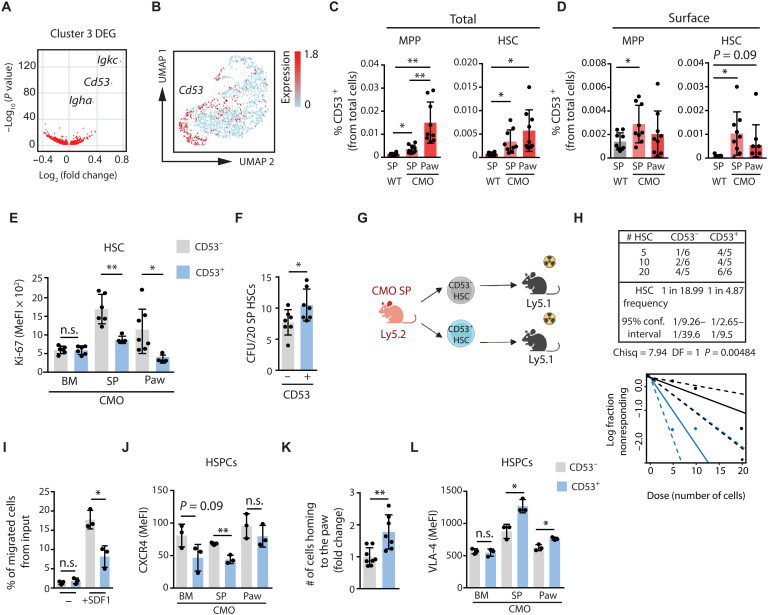
CD53 expression increases in a subset of extramedullary HSPCs with specific properties. (**A**) Volcano plot of the most differentially expressed genes in cluster 3. (**B**) UMAP plot displaying relative *Cd53* expression in all HSCs. (**C**) Frequency of total (intracellular and surface) CD53 MPP and HSC from WT spleen and CMO spleen and paw. The *y* axis indicates percentage from total cells. (**D**) Frequency of surface CD53^+^ MPPs and HSCs in WT spleen and CMO spleen and paw. The *y* axis indicates percentage of CD53^+^ cells from total cells. (**E**) Ki-67 levels in surface CD53^−^ and CD53^+^ HSCs from CMO BM, spleen, and paw. The *y* axis indicates median fluorescence intensity (MeFI). (**F**) HSC CFU quantification 10 days postplating. The *y* axis indicates CFU numbers. (**G**) Schematic of the transplantation setup. Created in BioRender. Alberich jorda, M. (2026) https://BioRender.com/cti22ub. (**H**) Frequency of functional surface CD53^−^ and CD53^+^ HSCs from CMO spleen measured by transplantation and calculated using ELDA software based on Poisson distribution statistics (chi-square test; Chisq = 7.94; *P* = 0.00484). Graph shows curve fit of the log fraction of nonresponding mice (solid lines) and confidence intervals (dashed lines) versus number of mice tested. The *x* axis indicates dose of transplanted cells, and the *y* axis percentages of nonresponders. A mouse was responder when PB engraftment was >0.01%, and at least two lineages reconstituted. Data show one representative experiment. (**I**) Percentage of HSPCs migrated toward no stimulation (−) or SDF-1α. Data shows one of the five representative experiments. (**J**) CXCR4 levels in HSPCs from CMO BM, spleen, and paw. (**K**) Number of splenic CMO HSPCs recovered 16 hours posttransplantation. Data are shown as fold change from surface CD53^−^ fraction. (**L**) VLA-4 levels in HSPCs. This figure includes 16- to 25-week-old male and female mice, except (I) to (L) used only female mice. Each symbol indicates values for one mouse. Two-tailed Student’s *t* test was used (*P < 0.05; **P < 0.01; n.s., not significant).

Next, we further explored the characteristics of HSCs isolated from CMO extramedullary sites. Examination of differentially expressed genes in extramedullary cluster 3 identified *Cd53* as a prominently up-regulated gene in HSCs from both spleen and paw of CMO mice (Fig. 4, A and B). We then examined total CD53 protein expression, including intracellular as well as extracellular membrane expression, in HSCs from WT and CMO BM, spleen, and paw by flow cytometry. This revealed an increased percentage of CD53^+^ HSCs based on total CD53 expression in CMO spleen and paw compared with WT spleen, supporting our Sort-seq results (Fig. 4C). Similarly, the percentage of CD53^+^ MPPs was increased in extramedullary sites (Fig. 4C). On the contrary, we did not observe increased percentage of CD53^+^ HSCs or MPPs based on total CD53 expression in CMO BM compared with WT BM (fig. S4, A and B), indicating that the elevation of CD53^+^ cells was restricted to the extramedullary sites. Because total CD53 expression does not necessarily correlate to the expression at the cell surface, we next assessed cell surface expression of CD53 on WT and CMO BM, spleen, and paw. We observed that the percentage of CD53^+^ HSCs and MPPs based on CD53 cell surface expression was not consistently up-regulated in extramedullary sites (Fig. 4D), possibly pointing at previously reported CD53 internalization and secretion upon activation (*29*–*31*). Further, the percentage of CD53^+^ HSCs and MPPs was not elevated in CMO BM compared with WT BM based on CD53 surface expression (fig. S4C). Next, we assessed the levels of Ki-67 proliferation marker in CD53^−^ and CD53^+^ HSCs, sorted according to CD53 surface expression, from distinct CMO tissues. While no differences were present between CD53^−^ and CD53^+^ CMO BM HSCs, CD53^+^ HSCs isolated from extramedullary sites exhibited reduced Ki-67 levels compared with their CD53^−^ HSC counterparts (Fig. 4E). This reduced Ki-67 expression in CD53^+^ HSCs correlated with a substantial increase of HSPCs in a G_0_ quiescent state (fig. S4, D and E). Together, we identified a unique set of HSPCs located at extramedullary sites expressing increased levels of CD53 and showing reduced signs of proliferation.

### Extramedullary CD53^+^ HSPCs exhibit distinct functional characteristics in comparison with CD53^−^ HSPCs

Next, we evaluated the colony-forming capacity of CD53^−^ and CD53^+^ HSCs, sorted according to CD53 surface expression and isolated from CMO spleen, using semisolid medium. Following a 10-day incubation, we observed that CD53^+^ HSCs exhibited an increased ability to form colonies in comparison with CD53^−^ HSCs (Fig. 4F). To assess the function of extramedullary CD53^−^ and CD53^+^ HSCs isolated from CMO spleen in vivo, cells were transplanted in extreme limiting dilution manner (Fig. 4G). Sixteen weeks upon transplantation, blood analysis demonstrated increased engraftment and hematopoietic reconstitution in recipient mice who received CD53^+^ spleen HSCs in comparison with recipients transplanted with CD53^−^ spleen HSCs (Fig. 4H and fig. S4, F and G). Because CD53 has been linked to immune cell migration (*31*–*33*), we investigated whether it similarly affects the HSPC migration toward Stromal Cell-Derived Factor-1 alpha (SDF-1α), a well-known HSPC chemokine. We observed that CD53^+^ HSPCs, sorted according to CD53 surface expression, migrated less toward SDF-1α than CD53^−^ HSPCs (Fig. 4I). Consistently, CD53^+^ HSPCs expressed lower levels of CXCR4 than CD53^−^ HSPCs (Fig. 4J). Furthermore, given the critical role of the CXCR4/SDF-1α signaling axis in the homing and retention of HSPCs within the BM, we next investigated the homing capacity of CD53^−^ and CD53^+^ HSPCs. While both cellular subsets homed similarly to BM and spleen of CMO mice (fig. S4H), we observed an increased homing to the CMO paws (Fig. 4K), suggesting that signals derived from this site strongly attract CD53^+^ HSPCs. Last, because Very Late Antigen-4 (VLA-4) anchors HSCs into protective BM niches and can interact with CD53 (*34*), we measured VLA-4 levels. We observed that CMO CD53^+^ HSPCs, based on CD53 surface expression, exhibit higher levels of VLA-4 in the spleen and paw (Fig. 4L), suggesting that HSPCs expressing CD53 on the surface might be specially adapted for docking and niche retention at inflamed extramedullary sites. Together, these findings show that surface CD53 marks a subset of extramedullary HSPCs with superior clonogenic and engraftment potential that preferentially homes to and is retained within inflamed peripheral niches possibly via elevated VLA-4, despite reduced CXCR4/SDF-1α–mediated migration.

### Extramedullary HSPCs exhibit up-regulation of immunosuppressive and MHCII-associated genes

**Fig. 5.**
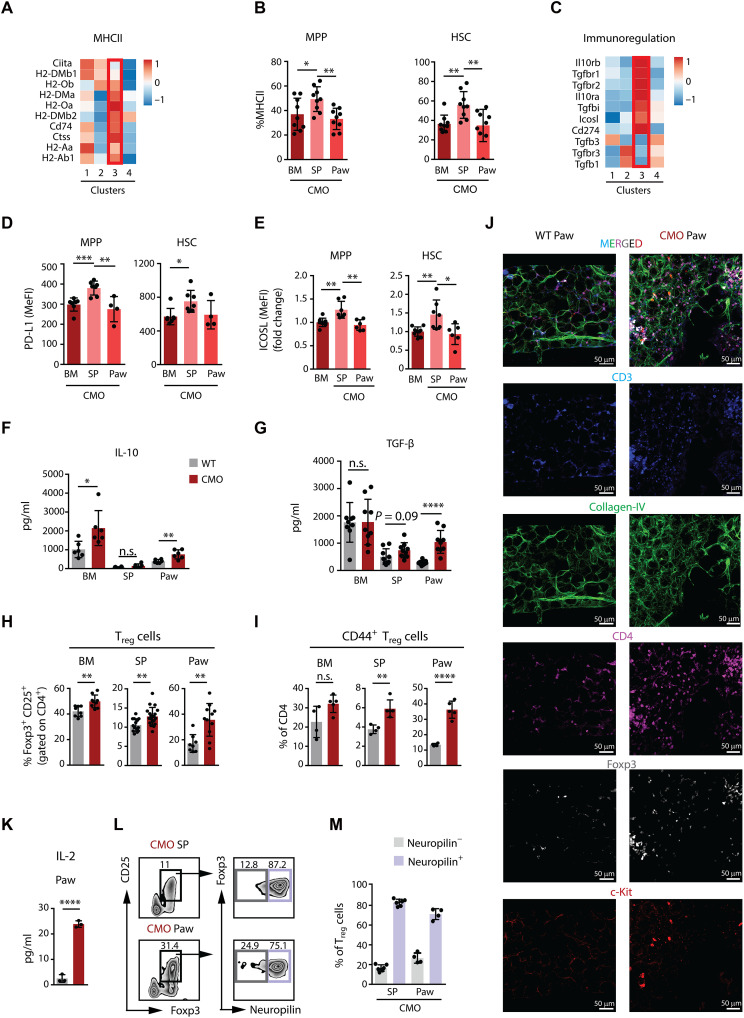
Extramedullary HSPCs express high levels of MHCII-associated and immunoregulatory genes and are surrounded by active T_reg_ cells. (**A**) Relative expression of genes associated with MHCII across the distinct HSC clusters. Red box highlights cluster 3, composed of extramedullary HSCs. (**B**) Frequency of MHCII^+^ MPP and HSC in CMO BM, spleen, and paw. The *y* axes indicate the percentage. (**C**) Relative expression of genes associated with immunoregulation across the distinct HSC clusters. Red box highlights cluster 3. (**D**) PD-L1 levels in MPP and HSC from CMO BM, spleen, and paw. The *y* axis indicates median fluorescence intensity (MeFI). (**E**) ICOSL levels in MPP and HSC from CMO BM, spleen, and paw. The *y* axis indicates MeFI. (**F**) IL-10 levels in WT and CMO BM, spleen, and paw. The *y* axis indicates units as picograms per milliliter. (**G**) TGF-β levels in WT and CMO BM, spleen, and paw. The *y* axis indicates units as picograms per milliliter. (**H** and **I**) Frequency of T_reg_ cells (H) defined as CD3^+^ CD4^+^ CD25^+^ Foxp3^+^ and activated CD44^+^ T_reg_ cells (I) in CMO BM, spleen, and paw. The *y* axes indicate percentage out of CD4^+^ cells. (**J**) Representative confocal images of WT and CMO paw showing Foxp3^+^ CD4^+^ T_reg_ cells in close proximity to c-Kit^+^ HSPCs, with CD3 (blue), collagen-IV (green), CD4 (magenta), Foxp3 (white), and c-Kit (red). (**K**) IL-2 levels in WT and CMO paw. The *y* axis indicates units as picograms per milliliter. (**L**) Gating strategy for Neuropilin^−^ (gray box) and Neuropilin^+^ (violet box) T_reg_ cells. The numbers indicate the percentages of parental gate. (**M**) Quantification of (L). Neuropilin^−^ T_reg_ cells (gray) and Neuropilin^+^ T_reg_ cells (violet) in CMO spleen and paw. The *y* axis indicates percentage of T_reg_ cells. In this figure, 16- to 25-week-old male and female mice were used. Each dot symbol indicates values for 1 mouse. Data indicate means ± SD from at least three independent experiments. Two-tailed Student’s *t* test was used to assess statistical significance (*P < 0.05; **P < 0.01; ****P < 0.0001; n.s., not significant).

Because our results suggest that extramedullary HSCs may exhibit properties distinct from those of HSCs located in BM, we further explored our transcriptomic data for unique features of extramedullary HSCs. First, we noticed that there was an up-regulation in the expression of genes implicated in antigen presentation via MHCII molecules in cluster 3 (Fig. 5A). Next, we observed that MHCII levels were up-regulated exclusively in HSCs and MPPs from CMO spleen (Fig. 5B and fig. S5, A and B). In addition to the up-regulation of MHCII, we noted enhanced expression of immunosuppressive regulatory genes, including genes coding for IL-10 receptor, transforming growth factor–β (TGF-β) receptor, Programmed Death-Ligand 1 (PD-L1) (CD274), and Inducible T-cell Co-stimulator Ligand (ICOSL) (Fig. 5, C to E). Further, we assessed the levels of classical costimulatory molecules CD80 and CD86, and we detected reduced expression in CMO spleen HSCs and MPPs (fig. S5C). The collective up-regulation of immunosuppressive and MHCII-associated genes in extramedullary HSPCs may indicate that these cells are trying to counteract the heavy inflammation by acquiring immunoregulatory properties.

### Extramedullary sites in CMO mice exhibit pro- and anti-inflammatory properties

To further characterize the environment in the inflammatory sites, we performed cytokine and chemokine profiling. We observed high levels of chemokines and proinflammatory cytokines in extracts from CMO paws (fig. S5D). Nevertheless, this was accompanied by an up-regulation of the anti-inflammatory cytokines IL-10 and TGF-β (Fig. 5, F and G). In addition, we noted a significant increase of T_reg_ cells across all CMO tissues (Fig. 5H), coupled with increased T_reg_ cell activation in the spleen and paws, as indicated by the CD44 marker (Fig. 5I). These EMH T_reg_ cells expressed all canonical T_reg_ cell markers (CD4^+^CD25^+^Foxp3^+^CD44^+^) at the same level as T_reg_ cells in healthy WT mice (fig. S5E), suggesting that chronic inflammation is not inducing development of a previously unidentified subset of T_reg_ cells, but rather expanding the already present T_reg_ cells. High-resolution confocal microscopy allowed us to detect the presence of T_reg_ cells in CMO paws, which were found in direct or close proximity to the c-kit^+^ cells (Fig. 5J and movies S3 and S4). Further, we found elevated IL-2 levels in CMO paws (Fig. 5K), cytokine which is known to enhance T cell proliferation and activation, especially in T_reg_ cells due to their high expression of the high-affinity IL-2 receptor CD25. Despite the increase of T_reg_ cell in CMO tissues, there was a noticeable overall decline in the frequencies of CD3^+^ T cells, affecting especially CD8^+^ T cells (fig. S5F). Additionally, a significant increase in the frequency of CD4^+^ T cells was observed in the paws of CMO mice, although no notable differences were found in the CD4^+^ populations within the BM and spleen (fig. S5F). Next, because T_reg_ cells can be induced peripherally, we assessed the expression of Neuropilin, which distinguishes thymic-derived T_reg_ cells (Neuropilin^+^) from those induced peripherally (Neuropilin^−^) (*35*). As anticipated, most of T_reg_ cells originated in the thymus, while a small fraction appeared to be generated on-site (Fig. 5, L and M). Collectively, our findings indicate that extramedullary HSPCs are influenced by a milieu of pro- and anti-inflammatory factors in an environment enriched for the presence of activated T_reg_ cells.

### Extramedullary CD53^+^ HSPCs promote T_reg_ cell development as a self-protective mechanism

**Fig. 6.**
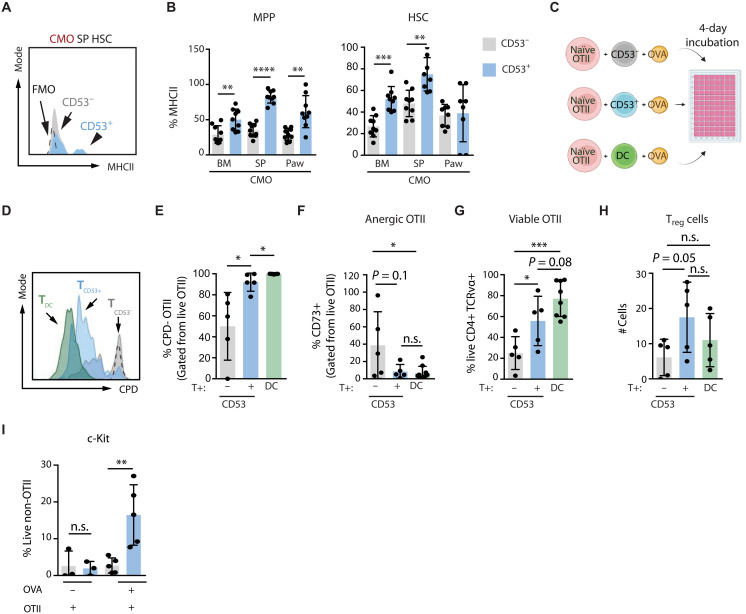
Mutual HSC/T_reg_ cell effects in extramedullary sites during chronic inflammation. In this figure, CD53^+^ and CD53^−^ HSPCs were sorted on the basis of CD53 surface expression. (**A**) Representative histogram plot of splenic HSCs from CMO mice. The *x* axis indicates MHCII levels in CD53^−^ and CD53^+^ HSCs. Gray peak indicates signal for fluorescence minus one (FMO). (**B**) Frequency of MHCII^+^ in CD53^−^ and CD53^+^ MPP and HSC from CMO BM, spleen, and paw. The *y* axes indicate percentage of MHCII^+^ cells. (**C**) Schematic representation of coculture experiments. (**D**) Representative histogram plot of T cell proliferation after 4 days in coculture with DCs (T_DC_; green), CD53^+^ HSPCs (T_CD53+_; blue), or CD53^−^ HSPCs (T_CD53−_; gray) in the presence of ovalbumin 247-264 peptide (OVA). The negative control (dashed line) represents naïve CD4^+^ OTII T cells and OVA without APCs. The *x* axis indicates levels of Cell proliferation dye (CPD). (**E**) Percentage of proliferated CD4^+^ OTII T cells after 4 days of coculture with CD53^−^ HSPCs (gray), CD53^+^ HSPCs (blue), and DCs (green) in the presence of OVA. (**F**) Percentage of anergic CD4^+^ OTII T cells after 4 days of coculture with CD53^−^ HSPCs (gray), CD53^+^ HSPCs (blue), and DCs (green) in the presence of OVA. (**G**) Frequency of viable CD4^+^ OTII T cells after 4 days of coculture with indicated cells in the presence of OVA. The *y* axis indicates percentage of viable CD4^+^ OTII cells. (**H**) Number of OTII T_reg_ cells after 4 days of coculture with the distinct cells types and in the presence of OVA. (**I**) Frequency of c-Kit^+^ HSPCs after 4 days of coculture with naïve CD4^+^ OTII cells. First two columns represent negative control cocultures containing HSPCs and naïve CD4^+^ OTII cells without OVA. The other columns represent cocultures containing HSPCs, naïve CD4^+^ OTII cells, and OVA. HSPCs were CD53^−^ (gray) or CD53^+^ (blue). Mice used were 16- to 25-week-old male and female mice. *P < 0.05; **P < 0.01; ***P < 0.001; and ****P < 0.0001; n.s.= not significant.

Next, we investigated whether MHCII up-regulation preferentially occurs in CD53^−^ or CD53^+^ HSPCs, separated on the basis of CD53 surface expression. We observed a significant increase of MHCII expression in CD53^+^ HSCs and MPPs compared with CD53^−^ counterparts (Fig. 6, A and B, and fig. S6A), and this positive correlation was also evident in other splenic populations (fig. S6, B and C). This observation suggests that HSPCs expressing CD53 on the cell surface may act as APCs and display antigens to T cells at the sites of inflammation, contributing to the development of peripherally derived T_reg_ cells in the spleen and paw of CMO mice (Fig. 5, L and M). To address this hypothesis, we sorted Lin^−^ c-kit^+^ CD11c^−^ CD53^+^ and Lin^−^ c-kit^+^ CD11c^−^ CD53^−^ HSPCs from CMO spleen and cocultured them with naïve T cells isolated from OTII transgenic mice in the presence of ovalbumin peptide (OVA) (Fig. 6C). After 4 days, CD53^+^ HSPCs showed a greater ability to activate naïve T cells compared with CD53^−^ HSPCs, as shown by the increased proliferation of T cells isolated from cocultures established with CD53^+^ HSPCs (Fig. 6, D and E) and by the low percentage of anergic T cells (Fig. 6F). Moreover, the viability of T cells was enhanced in the presence of CD53^+^ HSPCs in comparison with CD53^−^ HSPCs (Fig. 6G). Of note, the enhanced T cell proliferation and viability in CD53^+^ HSPC cocultures were similar to that obtained in the presence of professional APC dendritic cells (DCs; Fig. 6, D to G). The cocultures with CD53^+^ HSPCs generated a higher number of T_reg_ cells compared with cocultures with CD53^−^ HSPCs (Fig. 6H). Next, we investigated the effects of T cells generated in these cocultures on HSPCs themselves. We observed that CD53^+^ HSPCs exhibit a higher proportion of c-kit^+^ cells after 4 days of coculture with T cells in the presence, but not in the absence, of OVA, suggesting that their interaction with OVA-stimulated T cells in these cocultures preserves the stem and progenitor pool (Fig. 6I). Collectively, these results suggest that extramedullary HSPCs expressing surface CD53 may promote the proliferation and viability of T cells and enhance the development of T_reg_ cells to preserve the HSPC compartment at the sites of inflammation.

### Depletion of T_reg_ cells in CMO mice results in detrimental effects on splenic extramedullary HSCs

**Fig. 7.**
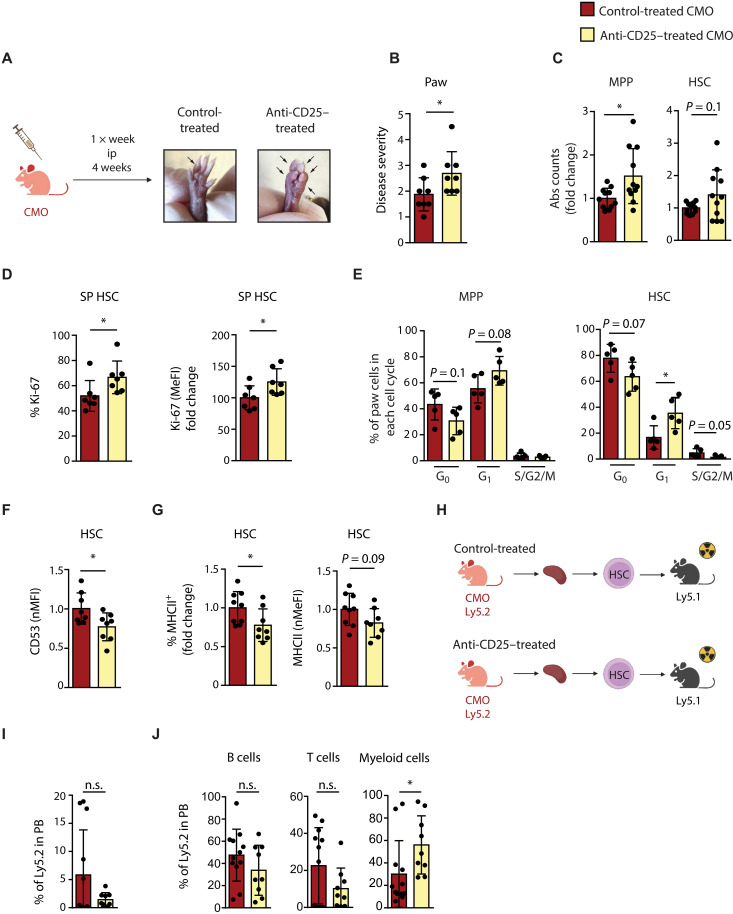
T_reg_ cell depletion in CMO mice further impairs HSCs. (**A**) Schematic representation of control and anti-CD25 treatment in CMO mice. Representative pictures illustrate CMO paws from control-treated (left) or anti-CD25–treated (right) mice. ip, intraperitoneal. Created in BioRender. Alberich jorda, M. (2026) https://BioRender.com/676os53. (**B**) Quantification of (A). The *y* axis indicates the degree of disease severity in paws from control-treated (red) or anti-CD25–treated (yellow) mice. (**C**) Absolute (Abs) number of MPP and HSC in CMO spleen. The *y* axes indicate the absolute counts as fold change from control-treated CMO. (**D**) Ki-67 expression in splenic HSC from control- and anti-CD25–treated CMO mice. The *y* axis indicates percentage (%) from parental HSC gate (left) and median fluorescence intensity (MeFI; right). (**E**) Cell cycle distribution of MPP and HSC in paw isolated from control- or anti-CD25–treated mice. (**F**) Total CD53 expression in splenic HSC from CMO mice, indicated as a fold change from nontreated CMO group. The *y* axis indicates CD53 normalized mean fluorescence intensity (nMFI). (**G**) Frequency of MHCII^+^ HSC (left) and MHCII levels (right) in CMO spleen. The *y* axis indicates percentage (%) from parental HSC gate (left) or indicates normalized median fluorescence intensity (nMeFI) for MHCII (right). (**H**) Schematic representation of the transplantation setup. Created in BioRender. Alberich jorda, M. (2026) https://BioRender.com/ciivoxz. (**I**) Engraftment of CMO splenic HSCs. The *y* axis indicates the percentage of donor-derived Ly5.2^+^ cells in PB. Nine to 10 recipients were used per group. (**J**) Trilineage reconstitution analysis in PB of recipient mice 16 weeks after transplantation. The *y* axes indicate the percentage of donor-derived B, T, and myeloid cells. In this figure, data indicate means ± SD from at least two independent experiments. All animals included in this figure were 16-week-old female CMO mice. Each dot indicates values for one biological sample. Two-tailed Student’s *t* test was used to assess statistical significance (*P < 0.05; n.s., not significant).

To further elucidate the role of T_reg_ cells on the HSPC compartment at inflammatory sites, we depleted T_reg_ cells in CMO mice using an anti-CD25 antibody (Fig. 7A and fig. S7A). A visual examination of the anti-CD25– and control-treated mice demonstrated that T_reg_ cell depletion enhanced the inflammatory phenotype of CMO mice, as observed by the degree of inflammation in paws (Fig. 7, A and B). Because the degree of inflammation in CMO mice correlates with the presence of neutrophils in the inflamed tissues (*36*, *37*), we assessed the number of neutrophils in the BM, spleen, and paw of treated mice. However, only a modest expansion in the absolute number of neutrophils was observed in the spleen of CMO mice upon anti-CD25 treatment (fig. S7A). Nevertheless, in line with the reduction of T_reg_ cells, we observed diminished IL-10 levels in the spleen and paw of anti-CD25 treated mice (fig. S7B). Next, we evaluated the abundance of MPPs and HSCs following the treatment of CMO mice. We noted increased numbers of HSPCs in spleen of anti-CD25–treated mice (Fig. 7C and fig. S7C), which could potentially be attributed to changes in proliferation (Fig. 7, D and E). Unexpectedly, the number of HSCs in paw of treated mice was not elevated, which could be related to the increased apoptosis of these cells (fig. S7, D and E). Additionally, total CD53 expression in spleen significantly decreased post–anti-CD25 treatment (Fig. 7F), while T_reg_ cell depletion did not change total CD53 expression in the BM and paw (fig. S7F). Further, we observed a reduction in MHCII levels in CMO spleen HSCs following anti-CD25 treatment (Fig. 7G), while no significant changes were recorded in the BM and paw (fig. S7G). Given our hypothesis that T_reg_ cells may exert a protective effect on extramedullary HSCs, we transplanted splenic HSCs isolated from anti-CD25– and control-treated CMO mice into lethally irradiated mice (Fig. 7H). The results indicated that untreated spleen HSCs tended to exhibit higher engraftment rates compared with their anti-CD25–treated counterparts (Fig. 7I). Furthermore, we observed a shift from lymphoid to myeloid bias in the anti-CD25–treated HSCs (Fig. 7J). Together, our data show that T_reg_ cell depletion amplifies local inflammation and drives the expansion of splenic HSCs characterized by low levels of total CD53, MHCII, and diminished engraftment capacity with a shift toward myeloid output, suggesting a critical, protective role for T_reg_ cells in sustaining the phenotype and function of extramedullary splenic HSCs.

## DISCUSSION

During the past decade, we have learned many aspects relevant to BM HSPC maintenance and fate, such as their location in the BM niche, transcriptomic profile, metabolic needs, and lineage bias. Nevertheless, the properties and hallmarks of HSPCs located outside of the BM remain less understood. In case of inefficient BM hematopoiesis, adult HSPCs can exit the BM niche and migrate to alternative hematopoietic sites, establishing EMH. For instance, extramedullary HSPCs can be detected in the spleen, kidney, and liver during benign hematological conditions and neoplastic disorders, as well as during inflammatory stress. Here, we use the CMO mouse model, characterized by the presence of sterile chronic inflammation, which demonstrated an abundance of circulating HSPCs in blood and the existence of EMH. We also report that, in addition to the classical tissues associated with EMH, nonclassical extramedullary sites, such as the inflamed tails and paws of CMO mice, can also host functional HSPCs. Previously, it was reported that myeloid progenitors can be accommodated in inflamed joints in mice suffering from systemic inflammation (*38*). In the present manuscript, using in vitro cultures as well as in vivo transplantation assays, we demonstrated that not only hematopoietic progenitors but also stem cells are present in inflamed tissues, where HSPCs and immune cells cohabit in an inflammatory environment and potentially influence each other. Because patients that exhibit EMH show ongoing hematopoiesis in classical as well as nonclassical extramedullary sites (*39*), we made use of our unique CMO murine model to investigate the properties of HSPCs in extramedullary sites and explore their relationship with the local inflammatory environment. Overall, our observations suggest that, as systemic IL-1β/IL-6–driven inflammation in CMO mice progressively disables BM hematopoiesis (*20*), consequently, a fraction of HSPCs may leave the marrow and seed peripheral tissues, thereby establishing EMH that temporarily rescues blood production. These same sites may soon become inflamed, yet the HSPCs up-regulate CD53, express MHC-II, acquire immunoregulatory properties, and act as APC, ultimately promoting T_reg_ cell differentiation, survival, and expansion. In exchange, T_reg_ cells suppress the cytokine excess, preserve HSPC quiescence, and maintain a small pool of functional HSPCs even within the hostile tissue.

We report and visualize the presence of HSPCs in the inflamed hind paws of CMO mice. Of note, based on the levels of HSPCs detected in CMO blood, we could rule out the possibility that the observed increase in paw HSPCs was due to residual blood contamination after perfusion. Accordingly, imaging of the CMO paw demonstrated that the HSPCs reside inside of the bone of the paws at extravascular locations, suggesting that chronic inflammation in CMO mice reactivates, otherwise, nonfunctional or “cold” BM regions in the paws, leading to renewed hematopoietic activity. This inflammation-driven reactivation of dormant BM niches could represent a specialized form of EMH, and future research should investigate whether other stress conditions, such as acute inflammation or cancer, can also induce this phenomenon.

In the present study, we profiled HSCs from WT BM and from CMO BM, spleen, and paw to identify unique HSC subpopulations and properties related to extramedullary HSCs. We determined that extramedullary HSCs share a similar gene expression profile marked by high expression of CD53, a member of the tetraspanin superfamily (*40*, *41*). Nevertheless, although spleen and paw CMO HSCs cluster together transcriptionally, they display clear niche-specific traits. Splenic HSPCs express higher levels of MHC-II, PD-L1, and ICOSL, which could sign a more tolerogenic/APC-oriented role, sampling blood-borne antigens and reinforcing systemic immune regulation. By contrast, paw HSPCs, reside inside the bone and exhibit a distinct phenotype, most likely influenced by the intense local inflammation. Nevertheless, the reason why these paw HSPCs present lower levels of MHCII, PD-L1, and ICOSL is yet to be elucidated. These baseline differences were mirrored when we depleted T_reg_ cells with anti-CD25 in CMO mice. In the spleen, this led to HSC expansion, a pronounced drop in CD53, and reduced stemness, as documented by our experiments and large body of evidence linking CD53 with quiescence as well as proliferation and HSC exhaustion (*42*–*46*). Nevertheless, we acknowledge that definitive proof of reduced stemness upon T_reg_ cell depletion can only be concluded from serial transplantation assays. In stark contrast, paw HSCs failed to expand and instead underwent more apoptosis, implying that T_reg_ cell loss in an already inflamed bone niche compromises survival rather than fuels proliferation. While the partial and variable T_reg_ cell depletion mediated by anti-CD25 administration limits our experimental model, these divergent outcomes highlight how extramedullary HSPC behavior may be differently influenced by the distinct sites. Future research exploring diverse strategies to deplete T_reg_ cells locally might provide a deeper understanding of extramedullary HSPC present at distinct locations.

While CD53 is uniquely expressed within the immune compartment, its role in HSC biology remains largely unknown. Recent studies have shown that CD53 expression is associated with a quiescent state in both human and murine HSCs (*44*, *47*). Accordingly, we observed that extramedullary CD53^+^ HSCs are less proliferative than CD53^−^ HSCs. Nevertheless, while Greenberg and colleagues reported that CD53 up-regulation induces quiescence in stressed HSCs through the DREAM complex, our single-cell RNA sequencing data did not show up-regulation of DREAM complex–related genes, pointing toward another mechanism responsible for the decreased proliferation observed in CD53^+^ HSCs. Alternatively, the involvement of the DREAM complex could be caused by the use of distinct models, ranging from endogenous CD53 expression to CD53 knockout mice (*44*), or by activation of the DREAM complex at the protein level despite no changes in RNA.

Previous studies have demonstrated that CD53 supports migration and mobilization of immune cells (*31*–*33*). In line with these reports, we observed that extramedullary HSPCs have up-regulated genes associated with cell migration and mobilization, such as *Selp*, *Sell*, and *Pecam1*. Functionally, CD53^+^ HSPCs behave as if the canonical BM retention pathway is attenuated. These cells express low levels of CXCR4 and migrate poorly toward SDF-1α, implying that the CXCR4/SDF-1α axis, critical for HSPC retention to the BM, is compromised in CD53^+^ HSPCs and may favor their exit from the CMO BM. Once in circulation, CD53^+^ HSPCs exploit a different adhesion system as they display high levels of VLA-4, and, after transplantation, they preferentially home to the inflamed paw. Because VLA-4 binds vascular cell adhesion molecule–1 (VCAM-1), a molecule up-regulated by inflammatory cytokines (*48*–*50*), we speculate that VCAM-1 is abundant in the paw and acts as a docking site. These observations allow us to argue that CD53 might fine-tune HSPC trafficking and retention within EMH niches. Nevertheless, it remains unknown whether these HSPCs migrate from the BM to the sites of inflammation or whether they are being produced at the sites of inflammation and are ready to migrate to other tissues. Our paired-paw analysis (inflamed hind paw versus noninflamed front paw) indicates that HSPCs appear only where local inflammation is present, supporting a model in which BM-derived cells home to and are retained within the inflamed tissue. Despite our observations, it remains unclear whether CD53 serves merely as a HSPC marker for a specific subset of cells or whether it has a direct functional role in HSPCs. Because total CD53 protein levels and cell surface expression do not correlate, likely due to receptor trafficking, internalization, and secretion in exosomes upon activation (*29*–*31*), we speculate that CD53 expression in HSPCs is not merely a marker but reflects a functional role in this compartment. At the same time, because much of our analysis relies on surface CD53, this discrepancy complicates data interpretation and suggests that surface levels may not fully capture the functional state of CD53 in HSPCs. Future studies integrating measurements of both surface and total CD53, including protein trafficking and signaling dynamics, will be needed to refine our understanding.

Our results provide discoveries that change our understanding of EMH and provide evidence for a mutual relationship between HSPCs and T cells at chronically inflamed extramedullary sites. We demonstrate that a subset of HSPCs located at inflammatory sites acts as APCs, promoting T cell proliferation, survival, and the development of T_reg_ cells, overall coordinating a mechanism trying to regulate inflammation at extramedullary sites. We observed that Pstpip2 deficiency in the hematopoietic compartment is necessary and sufficient to drive both systemic inflammation and EMH, while the contribution of the nonhematopoietic niche seems rather marginal. Nevertheless, we cannot exclude that the conditioning of the recipient mice used for transplantation affected and damaged the CMO niche and impaired its potential contribution. Together with the built-in front-versus-hind paw control, these data argue that local, myeloid cell–driven inflammation seeds extramedullary niches where the CD53 HSPCs and T_reg_ cells can cooperate. Notably, these extramedullary HSPCs were characterized by the elevated coexpression of CD53 and MHCII, molecules found in close proximity in the plasma membrane of human B cells and DCs (*51*). Previous studies have shown that antigen-presenting BM HSPCs can activate naïve CD4^+^ T cells (*52*). However, our findings indicate that extramedullary CD53^+^ HSPCs act as APCs and facilitate the production of T_reg_ cells. This is consistent with reports identifying a subpopulation of CD53^+^ megakaryocytes, which is able to activate T cells (*53*), altogether suggesting a general role for CD53 in APC regulation.

Not all T_reg_ cells in the inflamed sites were thymic-derived T_reg_ cells. EMH tissues are enriched in TGF-β, PD-L1, and ICOSL, known to favor peripheral T_reg_ cell induction and support a local origin for, at least, part of the T_reg_ cell pool. We suggest that CD53^+^ HSPCs contribute to the generation of these peripherally induced T_reg_ cells and speculate that this peripheral induction is an additional attempt to further modulate inflammation at the inflamed sites. In addition, we show that T_reg_ cells protect extramedullary HSPCs during chronic inflammation, preserving stem cell properties and supporting hematopoiesis at the extramedullary sites. While the expression of MHCII in all BM HSCs was previously reported (*52*), our results reveal that only a subset of extramedullary HSCs expresses MHCII. Mechanistically, we observe that MHCII expression in HSPCs triggers distinct immune responses in BM and extramedullary sites. Haas and colleagues reported that BM HSPCs interact with CD4^+^ T cells to promote HSPC proliferation, differentiation, and exhaustion of aberrant HSPCs during transformation, aiming to eliminate transformed HSPCs and prevent leukemia onset (*52*). In contrast, because BM hematopoiesis is inefficient under chronic inflammation (*20*), we propose that T_reg_ cells protect extramedullary HSCs, which support blood cell production at extramedullary sites. This HSC-protective mechanism is in line with previous reports highlighting the vulnerability of HSCs to excessive inflammatory signals (*7*, *15*).

Work by Haas and colleagues (*52*) showed that BM HSPCs naturally present self-peptides on MHC-II and can acquire exogenous antigens when exposed. Our study did not resolve the immunopeptidome of CMO HSPCs, but the specific pathogen-free nature of the colony indicates that dominant ligands are more likely to be self-derived peptides generated within the inflamed microenvironment. Nevertheless, we cannot exclude the contribution of opportunistic agents or microbiota-derived antigens, especially given prior evidence that microbial signals modulate the CMO phenotype (*37*).

The Pstpip2^L98P^ CMO mouse model serves as a useful tool to dissect how persistent, neutrophil IL-1β–driven inflammation reshapes the hematopoietic system and promotes EMH. Mutations in the *Pstpip1* gene are also associated with IL-1β–mediated autoinflammatory disorders. Because PSTPIP2 and PSTPIP1 mutations engage similar upstream pathways, insights gained here are likely to illuminate EMH in related human disorders, most notably CRMO and, with caution, PSTPIP1-mediated PAPA syndrome. Nevertheless, differences in cytokine bias and target tissues between these diseases mean that direct extrapolation must be made with caution. The CMO model informs about general mechanisms of inflammation-induced EMH but does not capture the full spectrum of clinical heterogeneity seen in human autoinflammatory diseases.

## MATERIALS AND METHODS

### Animals

Twelve- to 25-week-old CMO (Pstpip2^cmo/cmo^) and aged-matched C57BL/6J mice were used. Unless otherwise indicated in the figure legends, male and female mice were used in experiments. At this age, CMO mice displayed visible symptoms of the disease such as swollen paws, tail kinks, and developed EMH. OTII x Rag1 KO x Foxp3^DTR^ mice and OTII x Rag1 KO mice (used as fluorescence-minus-one controls) were culled at 6 to 12 weeks of age (*54*–*56*). For the transplantation assays, congenic strains Ly5.1 and Ly5.2 C57BL/6J were used. Mice were maintained under specific pathogen–free conditions in the animal facility of the Institute of Molecular Genetics of the CAS. Although routine microbiological screens excluded known pathogens, microbiota effects on the CMO phenotype were previously reported (*37*), presenting a potential limitation of the present study. All experiments were approved were approved by the Expert Committee on the Welfare of Experimental Animals of the Institute of Molecular Genetics and by the Czech Academy of Sciences and were in accordance with local legal requirements and ethical guidelines. All experiments were approved by the ethical committee of the institute (approval numbers AVCR 7141-2022 SOV II and AVCR 6654/2025 PPZ).

### Flow cytometric analysis and HSC sorting

WT and CMO mice were euthanized by cervical dislocation, long bones (femurs and tibias), spleen, and paws were isolated. Long bones and paws were processed to single-cell suspension by crunching using pestle and mortar. Spleens were processed using syringe pistons. Single-cell suspensions were filtered, and red blood cells were lysed using Ammonium-Chloride-Potassium buffer (ACK) for 5 min at room temperature. Next, cells were labeled with fluorescent-dye conjugated antibodies listed in table S2 and analyzed on Symphony instrument (BD Biosciences, San Jose, CA, USA).

For HSC sorting, tissues were processed as described above using a two-step method. Initially, the lineage positive fraction was tagged using biotinylated lineage-specific antibodies. These cells were then subjected to antibiotin magnetic beads separation (Miltenyi Biotec, Bergisch Gladbach, Germany), following the guidelines provided by the manufacturer using a magnetic-activated cell sorting separator. Subsequently, the lineage negative (Lin^−^) fraction was stained with various antibodies to label HSCs. An Influx instrument (BD Biosciences, San Jose, CA, USA) was used for sorting the HSCs using a defined strategy (Lin^−^, c-Kit^+^, Sca-1^+^, and CD48^−^ CD150^+^) (*57*). During sorting and flow cytometric analyses, either Hoechst 33258 or Zombie dye ultraviolet was included in the cell suspensions to identify and exclude nonviable cells. Antibodies were purchased from BD Biosciences (San Jose, CA, USA), eBioscience (San Diego, CA, USA), or BioLegend (San Diego, CA, USA) (see table S2). Data were obtained using Diva software (BD Biosciences, San Jose, CA, USA) and analyzed using FlowJo software (Tree Star Incorporation, Ashland, OR, USA).

### Blood transplantation assays

PB was collected from 15-week-old WT and CMO mice expressing Ly5.2. Red blood cells were lysed using the ACK buffer. Following the lysis, an equivalent of 800 μl was injected via tail vein into each lethally irradiated congenic mouse (Ly5.1) together with 0.5 × 10^6^ Ly5.1 support BM cells.

### Splenocyte transplantation assays

Spleens from Ly5.2 WT and CMO C57BL/6J mice were processed with syringe pistons to obtain single-cell suspensions. Splenocytes (1 × 10^6^) were counted and then mixed with Ly5.1 BM support cells (0.5 × 10^6^) before being intravenously transplanted into lethally irradiated Ly5.1 recipients. PB and BM analyses were performed 16 weeks posttransplantation. To distinguish between donor-derived and support cells, cells were labeled with antibodies against Ly5.1 and Ly5.2. Lineage-specific antibodies (B220, CD3, CD11b, and Gr1) were used to assess the reconstitution of B-cells (B220^+^), T cells (CD3^+^), and myeloid cells (CD11b Gr1^+^).

### Generation of BM chimeras

For BM chimeras, we used 16-week-old WT and CMO mice (Ly5.2) as donors. We isolated whole BM cells from their tibias and femurs. Three million whole BM cells were injected intravenously into lethally irradiated WT or CMO Ly5.1 recipient mice. Sixteen weeks later, the recipients were analyzed by flow cytometry.

### HSC extreme limiting dilution transplantation assays

For HSC limiting dilution transplantation assays, WT and CMO C57BL/6J mice expressing Ly5.2 were used, while congenic Ly5.1 C57BL/6J mice served as recipients. Donor mice used in these assays were typically 12 to 25 weeks old. Various doses of HSCs (*5*, *10*, *20*, *40*), characterized as LKS CD48^−^ CD150^+^, were sorted and then transplanted intravenously along with 0.5 × 10^6^ WT BM (Ly5.1^+^) support cells. Before transplantation, recipient mice underwent lethal irradiation. Analyses of PB and BM were conducted 16 weeks after transplantation. To differentiate between donor-derived and support cells, samples were labeled with antibodies against Ly5.1 and Ly5.2. Lineage-specific antibodies (B220, CD3, CD11b, and Gr1) were used to evaluate the reconstitution of B cells, T cells, and myeloid cells. A recipient mouse was considered successfully engrafted if a determined % of the cells were Ly5.2^+^ (figure legends indicate the specific cutoff in each experiment) and contributed to at least two of the three lineages assessed. The frequency of engrafted HSCs was determined using ELDA online software (*58*), which uses Poisson statistics and the method of maximum likelihood to estimate the proportion of negative recipients in a limiting dilution assay.

### Single-cell sort, sample preparation, and single-cell transcriptomic Sort-seq

One WT and six symptomatic CMO C57BL/6J mice were used for single-cell SORT-seq (*59*). BM HSCs from WT and CMO mice were isolated and sorted directly into 384-well microplates containing well-specific barcoded primers using an Influx instrument (BD Biosciences, San Jose, CA, USA). Spleen and paws from five CMO mice were processed, and their HSCs were also sorted directly into 384-well microplates. Spleen and paw cells from the five mice were pooled together, with each tissue type assigned to its own plate. Because the yield of WT splenic HSCs is too low, this population could not be included in the sequencing analysis. In total, WT BM HSCs were distributed across two plates and CMO BM HSCs across another two plates, and CMO spleen and paw HSCs were each allocated to a single plate. The plates were sealed and stored at −80°C before being shipped to Single Cell Discoveries (The Netherlands) for sequencing. Synthetic RNA control sequences (ERCC-00002 to ERCC-00171) were included in the sequenced libraries. The DNA libraries were paired-end sequenced on an Illumina NextSeq 500, high output, with a 1 × 75-base pair (bp) Illumina kit (read 1, 26 cycles; index read, 6 cycles; and read 2, 60 cycles). During sequencing, read 1 was assigned 26 bp and was used to identify the Illumina library barcode, unique molecular identifier (UMI; 6 bp), and cell barcode (8 bp). Read 2 was assigned 60 bp and used to map to the reference genome with STARSolo 2.7.10b. Mapping and generation of count tables were automated using the STARSolo 2.7.10b aligner.

### Single-cell Sort-seq analysis

Obtained reads were mapped to GRCm38 genome (Ensembl annotation version 98) (*60*). Quality control was performed to filter out low-quality cells. Cells with fewer than 1000 UMIs were excluded from further analysis. Cells with more than 10% of reads mapping to mitochondrial genes were removed to mitigate potential biases from stressed or dying cells. Secondary data analysis was conducted using BIOMEX software (*61*). Following quality control, data were normalized using log normalization and scaled. Contamination by other cell types, including B cells, was excluded on the basis of expression of lineage markers. Principal components analysis was performed on all features, followed by dimensionality reduction using Uniform Manifold Approximation and Projection (UMAP) in two-dimensional space. Graph-based clustering from the Seurat package (*62*) was applied to identify cell populations, resulting in five distinct clusters visualized on the UMAP plot. Ranked top markers from obtained clusters were acquired in a two-step manner. First, differential analysis was performed comparing each cluster against all other clusters where unique up-regulated markers were assigned, followed by ranking of genes using a product-based meta-analysis (*63*). In addition, we performed a pairwise differential analysis of cluster 4 versus all other clusters, and of HSCs isolated from CMO BM and WT BM, using limma package (*64*) and ranked the results by log_2_ fold change. Heatmap analysis was performed using heatmaply package (version 0.15.2) (*65*) with cluster-averaged gene expression data that have been autoscaled for visualization.

### Colony culture assays

Murine colony culture assays were performed using Methocult GF M3434 (STEMCELL Technologies, Vancouver, BC, Canada). Splenocytes (3 × 10^4^) and 100 μl of blood were plated after cell blood lysis using ACK. Paw cells (3 × 10^4^) and tail cells (3 × 10^4^) were sorted on the basis of viability and plated. For the CD53 HSC colony cultures, splenocytes from CMO mice were isolated and stained for Lin^−^, c-Kit^+^, Sca-1^+^, CD48^−^, and CD150^+^ along with CD53. HSCs were sorted as CD53^−^ and CD53^+^, and, subsequently, 20 CD53^−^ HSCs or 20 CD53^+^ HSCs were plated. In all cultures, colonies were counted and cells harvested after 7 to 10 days of in vitro culture. Pictures of the colonies were obtained on Apotome microscope.

### High-resolution microscopy

Hind paws from phosphate-buffered saline (PBS)–perfused WT and CMO mice aged 12 to 16 weeks were collected. Following removal of the epidermis, paws were fixed in 4% paraformaldehyde for 12 hours at 4°C. After two washes in PBS, samples were decalcified in OsteoSoft solution (Sigma-Aldrich) for 48 hours. Subsequently, the paws were washed three times with PBS and dehydrated in 30% sucrose for 2 to 3 days. Samples were then embedded in 5% low–gelling temperature agarose (Sigma-Aldrich), and 200- to 250-μm sections were obtained using a Leica vibratome. Sections were blocked and permeabilized for 12 hours at 4°C in 1 ml of staining buffer containing 0.2% Triton X-100 and 5% donkey serum in PBS. Samples were incubated with 500 μl of primary antibody mix diluted in staining buffer for 2 to 3 days at 4°C. Following primary antibody incubation, sections were washed three times for 2 hours each in wash solution (0.2% Triton X-100 in PBS). Secondary antibody staining was performed for 2 to 3 days in staining buffer, followed by three additional 2 hours washes in wash solution. Optical clearing was performed by incubating the sections in RapiClear 1.52 (SUNJin Lab) for 8 to 16 hours. Cleared paw slices were mounted on glass slides, surrounded by vacuum grease (Borer Chemie); excess RapiClear was removed by pipetting; and samples were sealed with coverslips. Reagents and antibodies used for microscopy are listed in table S3. Imaging was conducted using a Leica SP8 Stellaris confocal microscope equipped with a white light laser and a 405-nm diode laser. Large-area tile scans were stitched using Leica Application Suite X (LAS X) software. Three-dimensional visualization and postprocessing were performed using Imaris software (v10.1, Oxford Instruments). A 3 by 3 by 3 pixel median filter was applied to reduce pixel noise and aid manual cell annotation.

### Intracellular CD53 staining

To assess total CD53 expression in HSCs, cells from the BM, spleen, and paw were isolated and processed into a single-cell suspension, as described above. Subsequently, the cells were stained with HSC-specific surface markers (Lin^−^, c-Kit^+^, Sca-1^+^, and CD48^−^ CD150^+^) for 30 min at 4°C. After staining, the cells were washed twice with PBS containing 2% fetal bovine serum (FBS) and fixed using the BD Cytofix/Cytoperm kit (BD Biosciences) for 1 hour at 4°C. Following fixation, cells were washed with washing buffer and stained for CD53 according to the manufacturer’s instructions. Samples were analyzed by flow cytometry using a Symphony instrument (BD Biosciences, San Jose, CA, USA).

### Ki-67 staining in HSC

BM, spleen, and paw were isolated and processed into a single-cell suspension as described above. HSCs were stained using the following surface markers (Lin^−^, c-Kit^+^, Sca-1^+^, and CD48^−^ CD150^+^) for 30 min at 4°C. Next, the cells were washed twice with PBS containing 2% FBS and fixed using the BD Cytofix/Cytoperm kit (BD Biosciences) for 1 hour at 4°C. Following fixation, cells were washed and stained for Ki-67 for 30 min at 4°C according to the manufacturer’s instructions. Next, cells were washed again and stained with 4′,6-diamidino-2-phenylindole (75 ng per sample). Samples were analyzed by flow cytometry using a Symphony instrument (BD Biosciences, San Jose, CA, USA).

### Migration assays

Two thousand Lin^−^ c-Kit^+^ CD53^+^ or CD53^−^ cells were isolated from CMO BM as described above and resuspended in 100 μl of migration medium [1% bovine serum albumin in Iscove’s modified Dulbecco’s medium without fetal calf serum (FCS)]. Cells were seeded into 6.5-mm transwell inserts with 5.0-μm-pore polycarbonate membrane (Corning), precoated with bovine fibronectin (Sigma-Aldrich) at room temperature for 2 hours. The bottom chamber of the plate contained 600 μl of migration medium with or without murine recombinant SDF-1α (PeproTech) to a final concentration 100 ng/ml. Cells were left to migrate for 4 hours at 37°C in a CO_2_ incubator. Migrated cells were centrifuged and resuspended in 150 μl of bead suspension (SPHERO AccuCount Fluorescent Particles, 7.9-μm ACFP-70-10; 135 μl of PBS and 15 μl of beads) and measured on a BD Symphony flow cytometer (BD Biosciences, San Jose, CA, USA).

### Homing assays

Sixteen-week-old CMO mice were used as donors. We isolated whole BM cells from their tibias, femurs, and hips. The lineage positive fraction was labeled with biotinylated antibodies and subjected to antibiotin magnetic beads separation (Miltenyi Biotec, Bergisch Gladbach, Germany), following the manufacturer’s instructions. Separation was done using the Miltenyi LS columns (catalog number 130-042-401). After separation, the unlabeled fraction was stained and sorted on Influx. We sorted Lin^−^ c-Kit^+^ CD53^+^ and Lin^−^ cKit^+^ CD53^−^ cells and injected them into nonirradiated 8- to 10-week-old CMO recipients. Each recipient received 2000 to 5000 donor cells. The number of donor cells for the CD53^+^ and CD53^−^ fraction was the same in within each experiment.

### Flow cytometric analysis of T_reg_ cells

BM, spleen, and paw cells were isolated and processed as described above. First, cells were stained for 30 min at 4°C with surface markers CD3, CD4, CD8, and CD25 (to identify T cells) and with Neuropilin (to differentiate between thymic-derived and peripherally induced T cells). Subsequently, cells were washed twice with PBS containing 2% FBS and fixed/permeabilized using the eBioscience Foxp3/Transcription Factor Staining Buffer Set, according to the manufacturer’s instructions. After fixation and permeabilization, the Foxp3 antibody was added, and the cells were incubated overnight at 4°C. Next day, samples were washed and analyzed on a Symphony instrument (BD Biosciences, San Jose, CA, USA).

### Cocultures

HSPCs (Lin^−^ c-kit^+^ CD11c^−^ CD53^−^ and Lin^−^ c-kit^+^ CD11c^−^ CD53^+^) and DCs (CD11c^+^ MHCII^+^) were sorted into 96-well U-bottom plates. Each well contained 200 μl of RPMI supplemented with 10% heat-inactivated FCS (Gibco), penicillin/streptomycin (100 U/ml, Sigma-Aldrich), sodium pyruvate (1.5 mM, Gibco), and MEM nonessential amino acids (1×, Thermo Fisher Scientific). Before the cell sorting, OTII T cells (OTII x Rag1 KO x Foxp3^DTR^) were isolated from skin draining and mesenteric lymph nodes mechanistically using 40-μm cell strainer. Isolated cells were depleted of unwanted fraction using the CD4^+^ T cell Isolation Kit (Miltenyi) to enrich OTII T cells. Next, enriched OTII T cells were labeled with Cell Proliferation Dye eFluor 670 (eBioscience) according to the manufacturer’s protocol, stained for cell sorting, and sorted to the wells containing HSPCs or DCs in a ratio of 2000 HSPCs/DCs to 4000 naïve CD4^+^ OTII T cells. OVA peptide (OVA 323-339, InvivoGen) was introduced to stimulate the cocultures. As a negative control, OVA 257-264 was added to wells containing HSPCs/DCs and naïve CD4^+^ OTII T cells. Cells were incubated at 37°C and 5% CO_2_ for 4 days. T cell proliferation, T_reg_ cell numbers, and HSPC properties were assessed on the fourth day of coculture.

### Cytokine levels measurement

WT and CMO paws were crunched with a mortar and pestle in PBS, and supernatants were collected and used without freezing. Thirty-one cytokines, chemokines, and growth factors were measured using the Bio-Plex Pro Mouse Chemokine Panel 31-Plex #12009159 kit, according to the protocols of the manufacturer. Data acquisition was made using Bio-Plex 200 System (Luminex, Luminex Corporation, Technology Blvd, Austin Texas, USA) and software Bio-Plex Manager 6.0 (Bio-Rad Laboratories Inc., 2000 Alfred Nobel Drive, Hercules, CA, USA). Samples were measured under two distinct sensitivity modes [low reporter 1 (RP1) value for low sensitivity and high RP1 value for high sensitivity]. For the IL-10 and TGF-β measurements, WT and CMO BM, paws, and spleen were crunched with a mortar and pestle in PBS, and supernatants were collected and frozen. Total protein concentration was measured by Bicinchoninic Acid assay (Thermo Fisher Scientific, Waltham, Massachusetts, USA). The protein concentration-adjusted samples were measured using ELISA kits for the respective cytokines according to the manufacturer’s protocol (Thermo Fisher Scientific, Waltham, Massachusetts, USA).

### CMO mice anti-CD25 treatment

Twelve-week-old CMO mice were treated with InVivoMAb anti-mouse CD25 (IL-2Rα) (catalog number BE0012, BioXcell) over a period of 4 weeks. Mice received a weekly intraperitoneal injection of 0.4 mg per mouse. After 4 weeks, mice were euthanized, and their BM, spleen, and paws were harvested and processed into single-cell suspensions for flow cytometry analysis.

### Disease severity assessment

Disease severity in CMO mice was assessed by visually inspecting each hind paw, with each inflamed finger receiving a score of 0.5 points. Each cumulative score represents the total disease severity for an individual mouse.

### Statistical analysis

Statistical significance for indicated datasets was determined using two-sided, unpaired Student’s *t* test. *P* values of <0.05 were considered statistically significant. Poisson statistics and the method of maximum likelihood to estimate the proportion of negative recipients were used in extreme limiting dilution assays.
