# Supplementary material for: HSPCs and Treg cells cooperate to preserve extramedullary hematopoiesis under chronic inflammation
Source: Sci Adv. 2026 May 6;12(19):eadv9351. doi: 10.1126/sciadv.adv9351 (PMC13148345; doi:10.1126/sciadv.adv9351)
Supplement: Supplementary file 1 — Figs. S1 to S7 Tables S1 to S3 Legends for movies S1 to S4 [file sciadv.adv9351_sm.pdf]

Science Advances
AAAS

Supplementary Materials for

HSPCs and T_{reg} cells cooperate to preserve extramedullary hematopoiesis under chronic inflammation

Maria Kuzmina *et al.*

Corresponding author: Meritxell Alberich-Jorda, alberich@img.cas.cz

Sci. Adv. **12**, eadv9351 (2026)
DOI: 10.1126/sciadv.adv9351

The PDF file includes:

Figs. S1 to S7
Tables S1 to S3
Legends for movies S1 to S4

Other Supplementary Material for this manuscript includes the following:

Movies S1 to S4

Figure S1

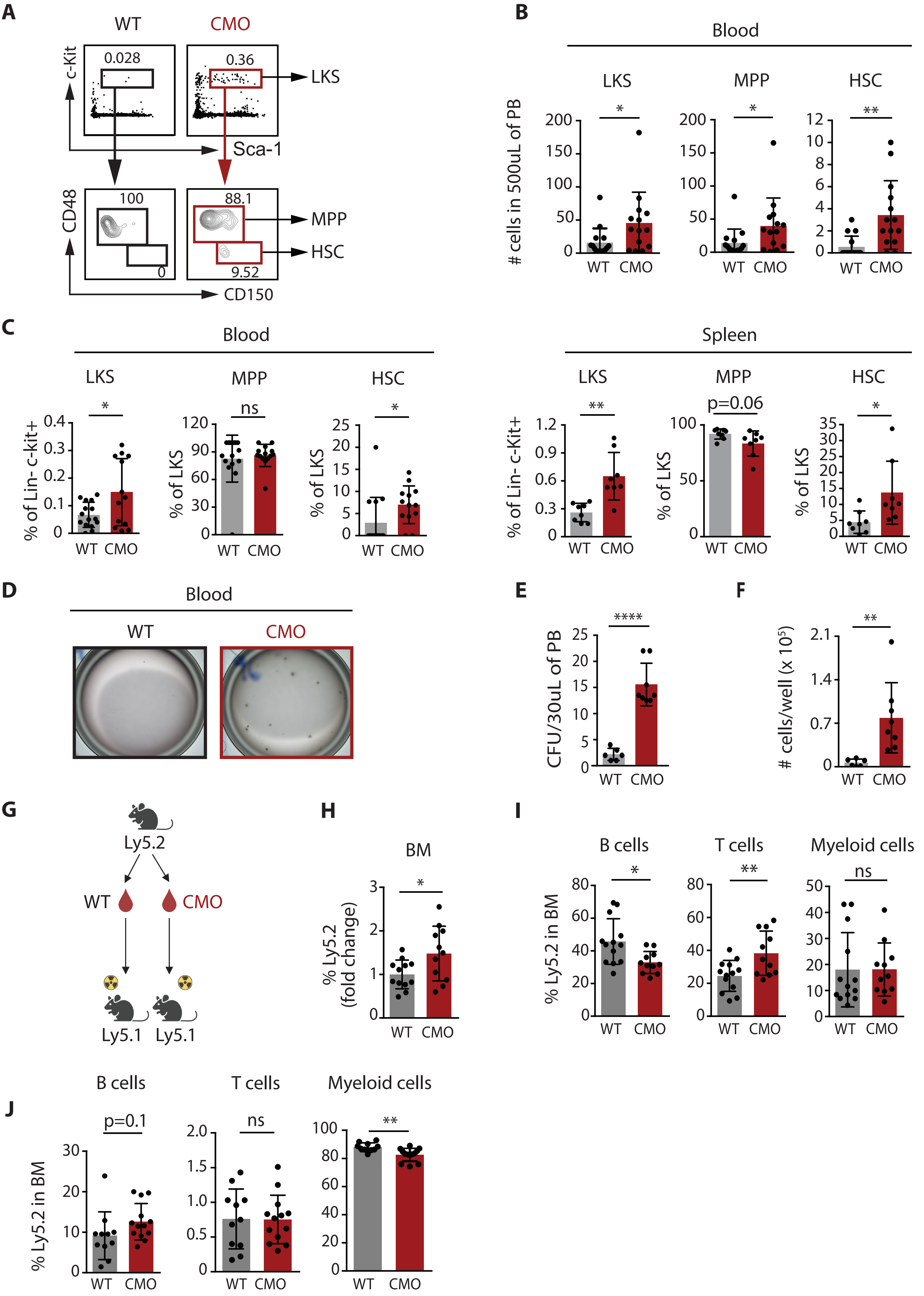

A
WT
CMO
c-Kit
0.028
0.36
LKS
Sca-1
CD48
100
0
88.1
9.52
MPP
HSC
CD150
B
Blood
LKS
MPP
HSC
cells in 500uL of PB
C
Blood
Spleen
% of Lin- c-kit+
% of LKS
ns
p=0.06
D
E
CFU/30uL of PB
F
cells/well (x 10^5)
G
Ly5.2
Ly5.1
H
BM
% Ly5.2 (fold change)
I
B cells
T cells
Myeloid cells
% Ly5.2 in BM
J
p=0.1

Figure S1. CMO mice exhibit increased numbers of functional HSPCs in peripheral blood.

(A) Representative flow cytometry plots from blood isolated from 1 WT and 1 CMO mouse. Upper plots illustrate c-Kit and Sca-1 expression in lineage- (Lin-) cells and the rectangle gates for Lin- c-Kit+ Sca-1+ (LKS) cells. Lower plots indicate CD48 and CD150 expression in LKS, and boxes illustrate gating for LKS CD48+ CD150- cells (MPPs) and LKS CD48- CD150+ cells (HSCs). Numbers indicate percentage from parental gates.

(B) Quantification of panel a. Y-axes indicate the number of LKS, MPP, and HSC in 500 µL of peripheral blood (PB) from WT (gray) and CMO (red) mice. At least 8 mice were included per group, and each mouse is represented by a dot symbol.

(C) Frequency of LKS, MPP, and HSC in blood and SP from WT (gray) and CMO (red) mice. Y-axes indicate percentage (%) from parental gate. Each dot indicates values for 1 mouse. All animals included were 16 to 20 weeks old. Data indicate mean ± SD from at least 2 independent experiments, and 2-tailes Student t test was used to assess statistical significance (*P , 0.05, **P , 0.01).

(D) Representative microscopy images of colony culture assays using MethoCult M3434. A total of 30 µL of PB from WT (gray) and CMO (red) mice were plated per well. Images correspond to day 7 of culture.

(E-F) Number of colony-forming units (CFU) (E) and cells (F) enumerated in panel c. Y-axes indicate the numbers per well at day 7. PB from at least 6 mice in 3 independent experiments was used in each condition. Each mouse is represented by a dot symbol.

(G) Schematic representation of blood transplantation assays. Cells present in 1000 µL of PB from WT or CMO mice (Ly5.2) were transplanted into lethally irradiated Ly5.1 recipient mouse along with 0.5x106 BM support cells (Ly5.1).

(H) Quantification of engraftment 16 weeks post-transplantation. Y-axes indicate percentage of WT (gray) and CMO (red) donor-derived Ly5.2+ cells in PB and BM. Engraftment is indicated as fold change from WT group. At least 11 animals were included in each group. Each dot indicates values for 1 animal.

(I) Lineage reconstitution analysis 16 weeks after transplantation in BM from recipients transplanted with blood from WT (gray) and CMO (red) mice. Y-axis indicates the percentage of donor-derived Ly5.2+ B cells, T cells, and myeloid cells. Each dot indicates values for 1 recipient mouse. At least 11 recipients were used in each group.

(J) Lineage reconstitution analysis 16 weeks after transplantation in BM from recipients transplanted with splenocytes from WT (gray) and CMO (red) mice. Y-axis indicates the percentage of donor-derived Ly5.2+ B cells, T cells, and myeloid cells. Each dot indicates values for 1 recipient mouse. At least 11 recipients were used in each group.

All animals included in Figure 1 were 12 to 25 weeks old. Data indicate mean ± SD from at least 3 independent experiments, and 2-tailed Student t test was used to assess statistical significance (*P , 0.05, **P , 0.01, ****P , 0.0001, and ns, not significant).

Figure S2

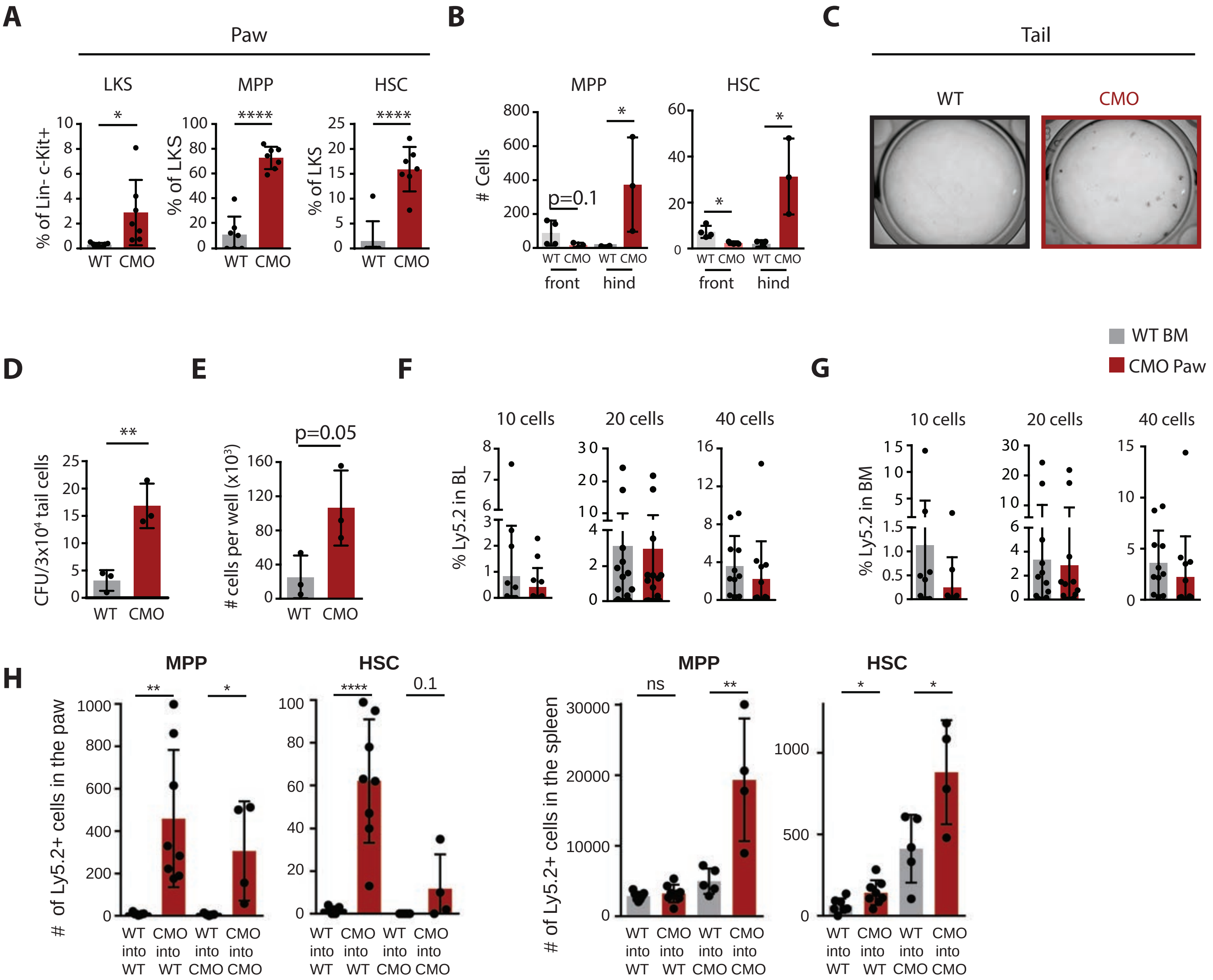

Figure S2. Presence of functional HSPCs in inflamed paws and tails.

(A) Frequency of LKS (Lin- c-Kit+ Sca1+), MPP (Lin- c-Kit+ Sca1+ CD48+CD150-) and HSC (Lin- c-Kit+ Sca1+ CD48-CD 150+) from paw. Y-axis indicates percentage (%) from parental gate in paw from WT (gray) and CMO (red) mice. Each dot indicates values for 1 mouse. All animals included were 16 to 20 weeks old. Data indicate mean ± SD from at least 3 independent experiments, and 2-tailed Student t test was used to assess statistical significance (*P , 0.05, ****P , 0.0001).

(B) Absolute number of MPP and HSC in front and hind paws. Y-axes indicate the number of MPP and HSC in cell suspensions from WT (gray) and CMO (red) paws. At least 3 mice were included per group in 2 independent experiments, and each mouse is represented by a dot symbol.

(C) Representative microscopy images of colony culture assays after 7 days of culture. 3x103 WT (gray) and CMO (red) cells from tail were plated per well using MethoCult M3434.

(D) Enumeration of panel b. Number of colony forming units (CFU) from cells isolated from WT (gray) and CMO (red) tails. Each dot symbol indicates values for one mouse. 2-tailed Student t test was used to assess statistical significance (**P , 0.01).

(E) Number of cells in cultures from panel b. Y-axes indicate numbers of cells per well at day 7. Tail cell suspensions from 3 mice were used in each condition.

(F and G) Quantification of engraftment in BL (F) and BM (G) 16 weeks post-transplantation. Y-axes indicate percentage of WT BM (gray) and CMO paw (red) donor-derived Ly5.2+ cells. Each dot indicates values for 1 animal.

(H) Presence of EMH Ly5.2+ MPPs and HSCs in paw (left panels) and spleen (right panels) of the generated chimeras. WT (gray) or CMO (red) BM were transplanted into Ly5.1+ WT or CMO recipients. Analysis was performed 16 weeks post-transplantation

Figure S3

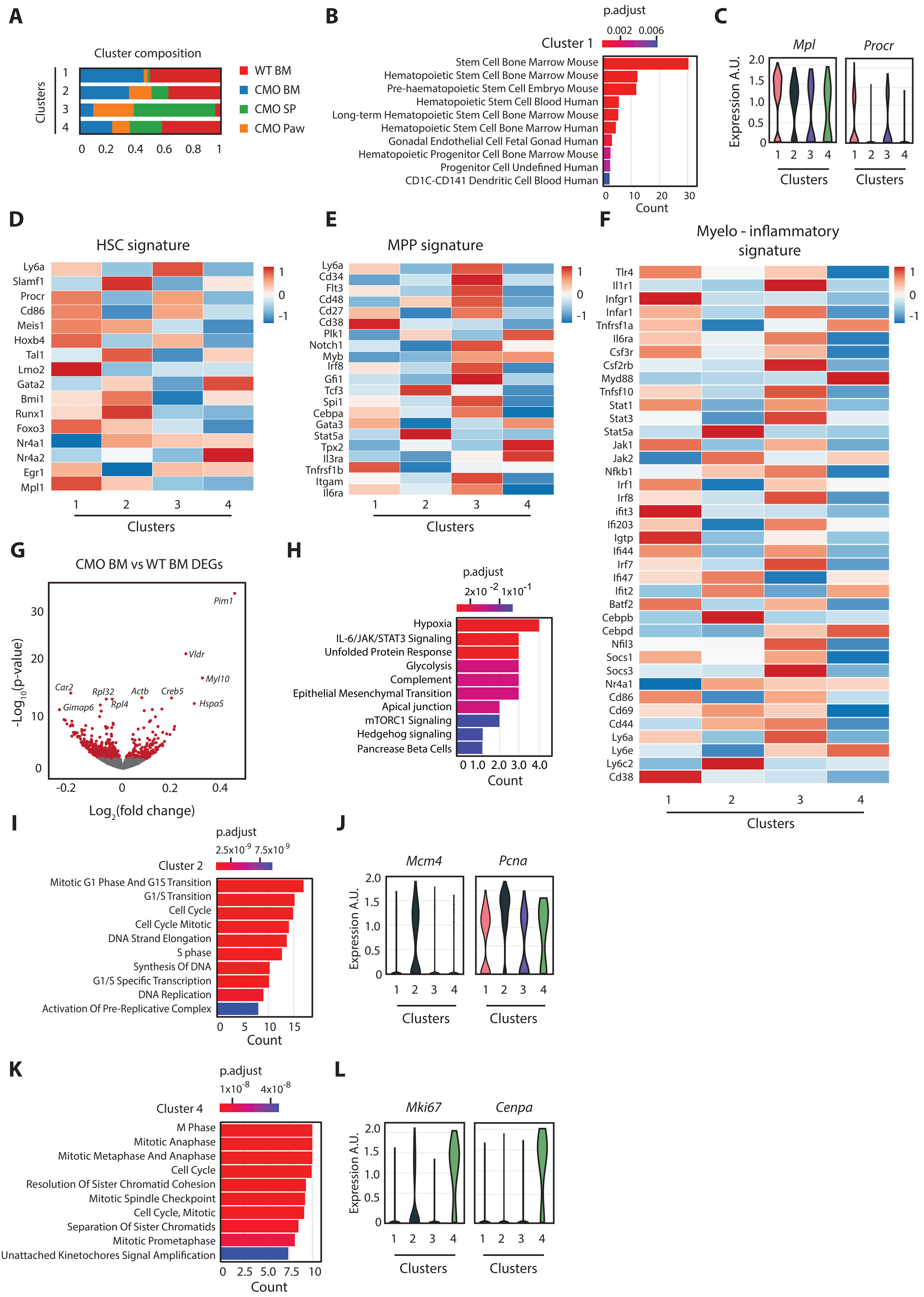
A
Cluster composition
Clusters
1
2
3
4
0 0.2 0.4 0.6 0.8 1
WT BM
CMO BM
CMO SP
CMO Paw
B
p.adjust
0.002 0.006
Cluster 1
Stem Cell Bone Marrow Mouse
Hematopoietic Stem Cell Bone Marrow Mouse
Pre-haematopoietic Stem Cell Embryo Mouse
Hematopoietic Stem Cell Blood Human
Long-term Hematopoietic Stem Cell Bone Marrow Mouse
Hematopoietic Stem Cell Bone Marrow Human
Gonadal Endothelial Cell Fetal Gonad Human
Hematopoietic Progenitor Cell Bone Marrow Mouse
Progenitor Cell Undefined Human
CD1C-CD141 Dendritic Cell Blood Human
0 10 20 30
Count
C
Mpl
Procr
Expression A.U.
2.0 1.5 1.0 0.5 0
1 2 3 4
Clusters
D
HSC signature
Ly6a
Slamf1
Procr
Cd86
Meis1
Hoxb4
Tal1
Lmo2
Gata2
Bmi1
Runx1
Foxo3
Nr4a1
Nr4a2
Egr1
Mpl1
1 0 -1
1 2 3 4
Clusters
E
MPP signature
Ly6a
Cd34
Flt3
Cd48
Cd27
Cd38
Plk1
Notch1
Myb
Irf8
Gfi1
Tcf3
Spi1
Cebpa
Gata3
Stat5a
Tpx2
Il3ra
Tnfrsf1b
Itgam
Il6ra
1 0 -1
1 2 3 4
Clusters
F
Myelo - inflammatory signature
Tlr4
Il1r1
Infgr1
Infar1
Tnfrsf1a
Il6ra
Csf3r
Csf2rb
Myd88
Tnfsf10
Stat1
Stat3
Stat5a
Jak1
Jak2
Nfkb1
Irf1
Irf8
ifit3
Ifi203
Igtp
Ifi44
Irf7
Ifi47
Ifit2
Batf2
Cebpb
Cebpd
Nfil3
Socs1
Socs3
Nr4a1
Cd86
Cd69
Cd44
Ly6a
Ly6e
Ly6c2
Cd38
1 0 -1
1 2 3 4
Clusters
G
CMO BM vs WT BM DEGs
Pim1
Vldr
Myl10
Car2
Rpl32
Actb
Creb5
Gimap6
Rpl4
Hspa5
$-\mathrm{Log}_{10}$(p-value)
30 20 10 0
-0.2 0 0.2 0.4
Log_2(fold change)
H
p.adjust
2×10^{-2} 1×10^{-1}
Hypoxia
IL-6/JAK/STAT3 Signaling
Unfolded Protein Response
Glycolysis
Complement
Epithelial Mesenchymal Transition
Apical junction
mTORC1 Signaling
Hedgehog signaling
Pancrease Beta Cells
0 1.0 2.0 3.0 4.0
Count
I
p.adjust
2.5×10^{-9} 7.5×10^{-9}
Cluster 2
Mitotic G1 Phase And G1S Transition
G1/S Transition
Cell Cycle
Cell Cycle Mitotic
DNA Strand Elongation
S phase
Synthesis Of DNA
G1/S Specific Transcription
DNA Replication
Activation Of Pre-Replicative Complex
0 5 10 15
Count
J
Mcm4
Pcna
Expression A.U.
2.0 1.5 1.0 0.5 0
1 2 3 4
Clusters
K
p.adjust
1×10^{-8} 4×10^{-8}
Cluster 4
M Phase
Mitotic Anaphase
Mitotic Metaphase And Anaphase
Cell Cycle
Resolution Of Sister Chromatid Cohesion
Mitotic Spindle Checkpoint
Cell Cycle, Mitotic
Separation Of Sister Chromatids
Mitotic Prometaphase
Unattached Kinetochores Signal Amplification
0 2.5 5.0 7.5 10
Count
L
Mki67
Cenpa
Expression A.U.
2.0 1.5 1.0 0.5 0
1 2 3 4
Clusters

Figure S3. Identification and classification of distinct HSCs clusters.
(A) Relative contribution of WT BM, CMO BM, CMO SP and CMO paw HSCs to the cellular composition of each cluster. Y-axis indicates the cluster number. X-axis indicates the relative contribution of the individual HSC samples to each cluster.
(B) Relevant results of enrichment analysis of differentially expressed genes by Enrichr in cluster 1.
(C) Violin plots representing the expression of Mpl and Procr genes in cluster 1.
(D) Gene expression level of genes defining a HSC signature in each cluster.
(E) Gene expression level of genes defining a MPP signature in each cluster.
(F) Gene expression level of genes defining a myelo-inflammatory signature in each cluster.
(G) Volcano plot showing the differentially up- and downregulated genes in HSCs isolated from CMO BM and WT BM.
(H) Relevant results of enrichment analysis of differentially expressed genes by Enrichr in CMO BM HSCs.
(I) Relevant results of enrichment analysis of differentially expressed genes by Enrichr in cluster 2.
(J) Violin plots representing the expression of Mcm4 and Pcna genes in cluster 2.
(K) Relevant results of enrichment analysis of differentially expressed genes by Enrichr in cluster 4.
(L) Violin plots representing the expression of Mki67 and Cenpa genes in cluster 4.

Figure S4

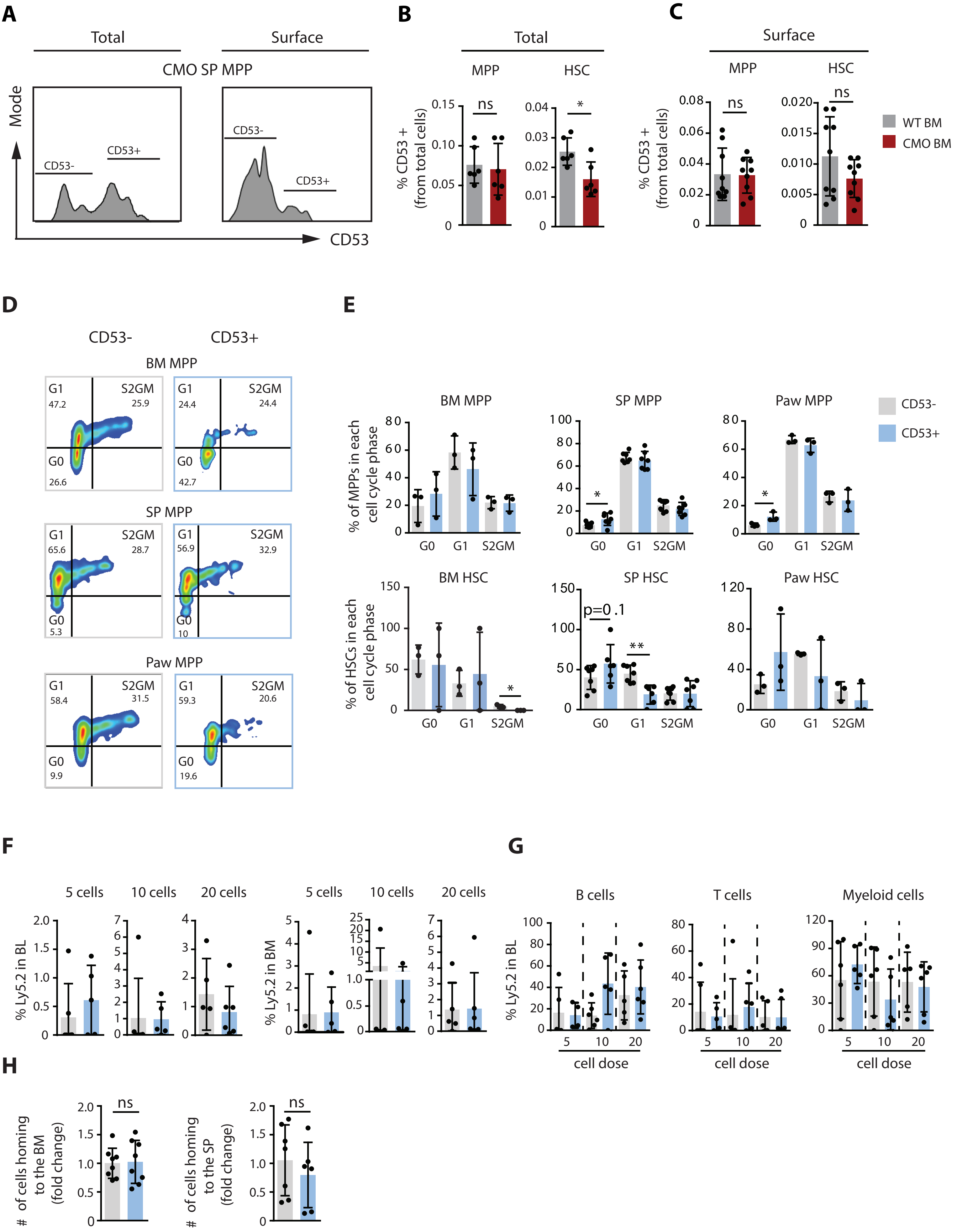
A
Total
Surface
CMO SP MPP
Mode
CD53-
CD53+
CD53
B
Total
MPP
HSC
% CD53 + (from total cells)
ns
*
C
Surface
MPP
HSC
ns
ns
WT BM
CMO BM
D
CD53-
CD53+
BM MPP
SP MPP
Paw MPP
G1
S2GM
G0
E
BM MPP
SP MPP
Paw MPP
BM HSC
SP HSC
Paw HSC
% of MPPs in each cell cycle phase
% of HSCs in each cell cycle phase
CD53-
CD53+
p=0.1
**
F
5 cells
10 cells
20 cells
% Ly5.2 in BL
% Ly5.2 in BM
G
B cells
T cells
Myeloid cells
cell dose
H
# of cells homing to the BM (fold change)
# of cells homing to the SP (fold change)
ns

Figure S4. CD53 expression and engraftment of CD53+ and CD53- HSCs.
(A) Representative histogram plots of total and surface CD53 in CMO SP MPP. Lines indicate CD53- and CD53+ MPP.
(B) Frequency of total (intracellular + surface) CD53+ MPPs and HSCs in WT and CMO BM. Y-axis indicates percentage (%) of CD53+ cells from total cells.
(C) Frequency of surface CD53+ MPPs and HSCs in WT and CMO BM. Y-axis indicates percentage (%) of CD53+ cells from total cells.
(D) Representative flow cytometry plots. Cell cycle distribution based on DAPI and Ki-67 staining. MPP from BM, SP, and paw were divided into CD53- and CD53+ based on surface expression.
(E) Quantification of cell cycle distribution based on staining shown in panel D. Each dot indicates values for 1 animal. In this experiment only male mice were used.
(F) Quantification of CD53- and CD53+ CMO SP HSCs engraftment 16 weeks post-transplantation. Number of cells transplanted is indicated. Y-axes indicate percentage of donor-derived Ly5.2+ cells in blood (BL, left panel) and BM (right panel). Each dot indicates values for 1 animal.
(G) Quantification of CD53- and CD53+ CMO SP HSCs lineage reconstitution 16 weeks post-transplantation. Number of cells transplanted is indicated. Y-axes indicate percentage of donor-derived Ly5.2+ cells in PB. Each dot indicates values for 1 animal.
(H) Absolute number of CD53- (gray) and CD53+ (blue) Ly5.2+ cells that homed to the BM (left panel) or to the spleen (SP) overnight. Y-axes indicate the number as fold change from CD53- HSPCs. In this exper-iment only females were used. Each dot indicates values for 1 animal.

Figure S5

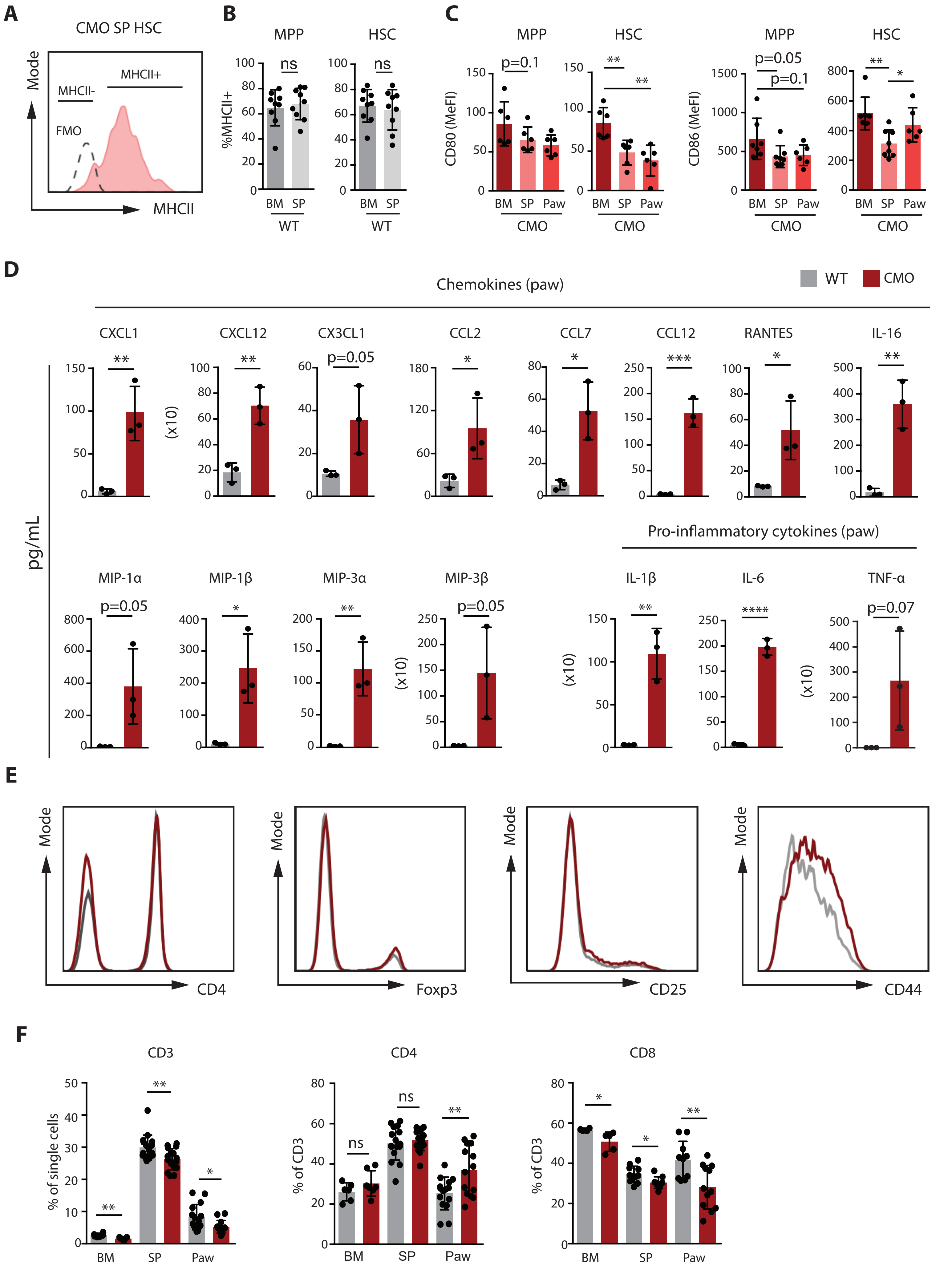
A
CMO SP HSC
Mode
MHCII-
MHCII+
FMO
MHCII
B
MPP
HSC
ns
%MHCII+
BM SP
WT
C
MPP
HSC
p=0.1
CD80 (MeFI)
BM SP Paw
CMO
p=0.05
p=0.1
CD86 (MeFI)
D
Chemokines (paw)
WT
CMO
CXCL1
CXCL12
CX3CL1
CCL2
CCL7
CCL12
RANTES
IL-16
pg/mL
(x10)
p=0.05
Pro-inflammatory cytokines (paw)
MIP-1α
MIP-1β
MIP-3α
MIP-3β
IL-1β
IL-6
TNF-α
p=0.07
E
Mode
CD4
Foxp3
CD25
CD44
F
CD3
CD4
CD8
% of single cells
% of CD3
BM
SP
Paw
ns

Figure S5. Pro- and anti-inflammatory environment in CMO paws.

(A) Histogram plot showing MHCII expression in CMO SP HSC. Lines indicate MHCII- and MHCII+ cells. Dotted line shows signal for FMO.

(B) Frequency of MHCII+ MPP and HSC in SP and BM from WT mice. Y-axes indicate the percentage (%) of MHCII positive cells.

(C) Expression of CD80 (left panel) and CD86 (right panel) in MPP and HSC isolated from CMO BM, SP, and paw. Y-axes indicate median fluorescence intensity (MeFI).

(D) Chemokine and cytokine profiling by BioPlex in CMO paw. Each dot symbol indicates values for 1 mouse. Data indicate mean ± SD from 1 independent experiments.

(E) Representative histogram plots showing expression of the indicated markers in WT splenic Tregs (gray) and CMO splenic Tregs (red).

(F) Frequency of CD3, CD4 and CD8 in BM, SP and paw HSCs from CMO mice. Y-axis indicates percentage (%) of CD3, CD4 or CD8 from parental gate. Each dot symbol indicates values for 1 mouse. Data indicate mean ± SD from 2 and more independent experiments.

All animals included were 16 to 25 weeks old. 2-tailed Student t test was used to assess statistical significance (*P , 0.05, **P , 0.01, ****P , 0.0001).

Figure S6

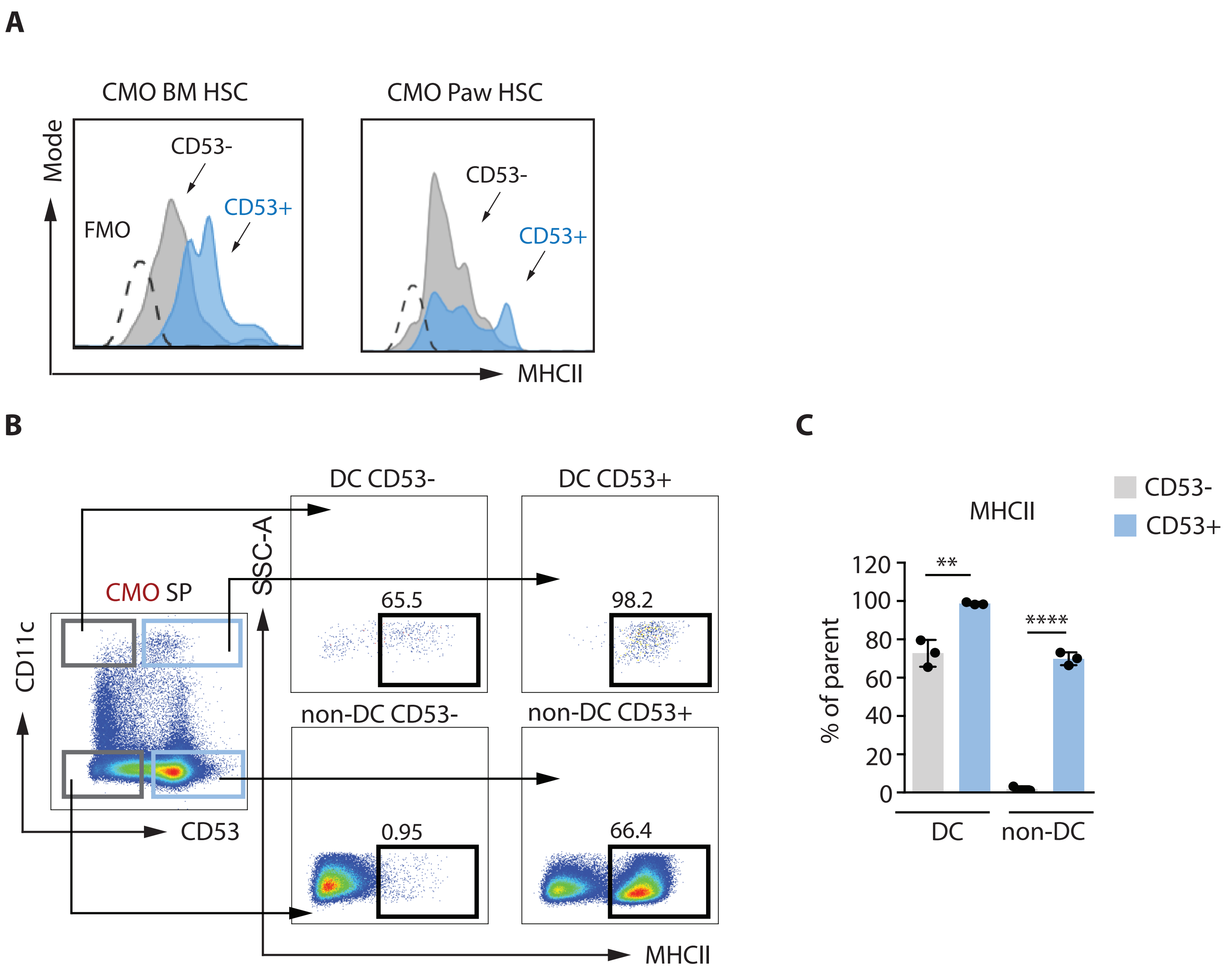

Figure S6. CD53+ cells contain higher levels of MHCII.
(A) Representative histogram plots showing MHCII expression. Arrows indicate a subset of CD53- and a CD53+ HSCs in CMO BM (left panel) and CMO paw (right panel).
(B) Representative gating strategy for MHCll in CMO SP. Splenocytes were gated according to CD11c and CD53 levels. Upper left box indi cates CD53- dentritic cells (DC CD53-), upper right box CD53+ CD11c+ cells (DC CD53+), lower left box CD53- CD11c- (non-DC CD53-), and lower right box CD53+ CD11c- (non-DC CD53+). The rest of the dot plots indicate MHCll levels in the distinct populations. Black boxes indicate MHCll+ cells. Numbers indicate percentages from parental gates.
(C) Frequency of MHCll expression across in DC and non-DC populations from CMO SP. CD53- cells are indicated in gray and CD53+ in blue. Each dot symbol indicates values for 1 mouse. Data indicate mean ± SD from 1 experiment. 2-tailed Student t test was used to assess statistical significance (**P , 0.01, ****P , 0.0001).

Figure S7

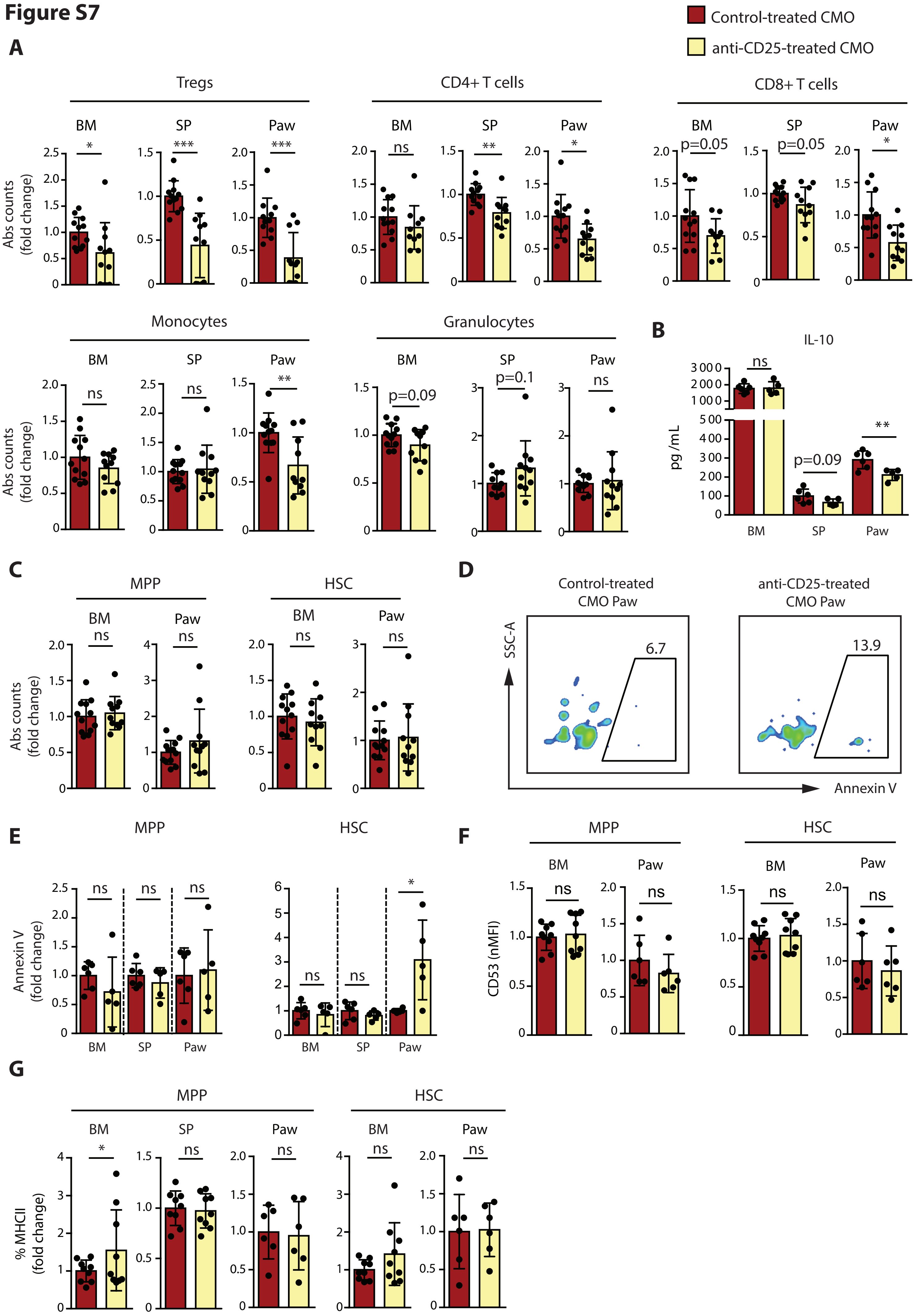

Control-treated CMO
anti-CD25-treated CMO
A
Tregs
CD4+ T cells
CD8+ T cells
BM
SP
Paw
Abs counts (fold change)
p=0.05
Monocytes
Granulocytes
p=0.09
p=0.1
B
IL-10
pg/mL
C
MPP
HSC
D
Control-treated CMO Paw
anti-CD25-treated CMO Paw
SSC-A
Annexin V
6.7
13.9
E
Annexin V (fold change)
F
CD53 (nMFI)
G
% MHCII (fold change)

Supplementary Figure 7. Treg depletion effect on extramedullary HSC and MPP.

(A) Absolute counts of Tregs, CD4, CD8, monocytes, and granulocytes in non-treated (red) and anti-CD25 treated (yellow) CMO mice in BM, SP and paw. Y-axis indicates absolute counts as fold change from non-treated (red) CMO group. X-axis indicates whether mice received (yellow) or not (red) anti-CD25 antibody treatment.

(B) IL-10 levels in BM, SP and paw of CMO mice non-treated (red) or treated (yellow) with anti-CD25 antibody. Y-axis indicates IL-10 levels as pg/mL.

(C) Absolute counts of MPP and HSC in non-treated (red) and anti-CD25 treated (yellow) CMO mice in BM and paw. Y-axis indicates absolute counts as fold change from non-treated (red) CMO group. X-axis indicates whether mice received (yellow) or not (red) anti-CD25 antibody treatment.

(D) Representative flow cytometry plots of paw HSC isolated from control-treated or anti-CD25 antibody-treated mice. X-axes indicate levels of annexin V.

(E) Quantification of panel D.

(F) Flow cytometric analysis of total CD53 expression in MPP and HSC from CMO BM and paw. Total CD53 expression is indicated as a fold change from non-treated (red) CMO group. Y-axis indicates CD53 mean fluorescence intensity (MFI). Each dot indicates values for 1 mouse.

(G) Frequency of MHCII+ MPP and HSC in BM, SP, and paw of non-treated (red) or anti-CD25-treated (yellow) mice. Y-axis indicates percentageas fold change from non-treated control.

In this figure, data indicate mean ± SD from 3 independent experiments. 2-tailed Student t test was used to assess statistical significance (*P , 0.05, **P , 0.01, ***P , 0.001, ns, not significant). Female CMO mice were used to perform anti-CD25 or control treatment.

Rank	Cluster 1	Cluster 2	Cluster 3	Cluster 4
1	Gm19590	Mcm6	Igkc	Ccnb2
2	Mpl	Pcna	Cd53	Cenpa
3	Myl10	Mcm5	Ly6a	Hmgb2
4	Itsn1	Lig1	Prtn3	Mki67
5	Hlf	Cdca7	Ifitm1	Hist1h2bc
6	Zbtb20	Dnmt1	Pim1	Ptms
7	Mmrn1	Mcm7	Shisa5	Cks2
8	Zfp950	Cdk6	Sox4	H2afz
9	Plcb2	Tuba1b	Adgrl4	Dynll1
10	Txnip	Mcm2	Znrf1	Cenpe
11	Ccnd2	Mcm3	Ikzf2	Ncapd2
12	Eif4a2	Dtl	Igha	Knstrn
13	Mecom	Nop56	Ypel3	H2afv
14	Tbxas1	Ybx3	Plppr3	Cenpf
15	Hacd4	Srm	Chd3	H1f0
16	Pdzk1ip1	Dut	Fut8	Hmgn2
17	Xbp1	Fam111a	Vldlr	Nde1
18	Dusp2	Cbx5	Akap13	Stmn1
19	Tgm2	Cdt1	Il2rg	Cdca8
20	Neat1	Rfc2	Pou2f2	Hp1bp3
21	Aldoa	Atad2	Gm2a	Lockd
22	Smpdl3a	Rrm1	Selp	Hmgb3
23	Nfat5	Dctpp1	Glul	Rad21
24	Ebi3	Ranbp1	Ifi203	Cdca3
25	Kit	Tfdp1	Apobec3	Bub1b
26	Gbp6	Mif	Plac8	Cbx3
27	Gimap8	Nme1	Rcsd1	Racgap1
28	Rbl2	Topbp1	Emb	Tpx2
29	Sult1a1	Top2a	Cd74	Hdgf
30	Rbm5	Uhrf1	Klhl24	Sun2
31	Pygm	Itga2b	Samsn1	Cd9
32	St3gal1	Slc25a5	Map4k4	Arcn1
33	Pnrc1	Mcm4	Trim30a	Hmgb1
34	Gtf2i	Atp5g1	Gramd1a	Usp47
35	Csad	Usp1	Gstm1	Lsm4
36	Limd2	Slbp	Snrnp70	Nucks1
37	Msi2	Cycs	Rsrp1	H1fx
38	Zfp467	Pa2g4	Retreg1	Paip2
39	Procr	Prim1	Lpp	Ctnnb1
40	Angpt1	Dhfr	Arid5a	Acsl5
41	Gimap6	Hells	Foxp1	Selenoh
42	Lyl1	Cox5a	Stat3	Sh3bgrl
43	Slc50a1	Gnl3	Trim30b	Ube2c
44	Pik3ip1	Ran	S100a4	Cox7b
45	Trpc6	Snrpd1	Mettl7a1	Ckap5
46	Smarca2	Smc6	Meg3	Tmpo
47	Eng	Tyms	Sell	Vbp1
48	Uba7	Slc22a3	Hbb-bt	Cep55
49	Mllt3	Dek	Ablim1	Clic4
50	Epb41l4b	Actn4	Dtx3l	Ptbp1

Table S1. List of top50 genes differentially expressed in the 4 identified clusters

Mouse antibodies	Source
Mouse Lineage Cocktail Pacific Blue (including CD3 (17A2); Gr1 (RB6-8C5); CD11b (M1/70); CD45R/B220 (RA3-6B2); TER-119 (Ter-119)	BioLegend
c-Kit PE (2B8)	BioLegend
c-Kit BUV395 (2B8)	BD Biosciences
c-Kit BV711 (2B8)	BioLegend
Sca-1 APC (D7)	BioLegend
Sca1 BV605 (D7)	BD Biosciences
CD48 FITC (HM48-1)	BioLegend
CD48 PE (HM48-1)	BioLegend
CD150 Pe-Cy7 (TC15-12F12.2)	BioLegend
CD53 Alexa Flour 647 (OX-79)	BD Biosciences
MHCII (I-A/I-E) FITC (M5/114.15.2)	BioLegend
MHCII (I-A/I-E) BUV737 (M5/114.15.2)	Biolegend
MHCII (I-A/I-E) PB (M5/114.15.2)	BioLegend
CD11c APC-Cy7 (N418)	Biolegend
CD73 PB (Ty/11.8)	Biolegend
TCR Vα2 PE (B20.1)	Biolegend
Hoechst 33258	Sigma- Aldrich
Fixable Viability dye eFluor 506	eBioscience
Ghost dye UV 450 Viability Dye	TONBO biosciences
B220 PE (RA3-6B2)	BioLegend
B220 APC (RA3-6B2)	BioLegend
CD11b PE (M1/70)	BioLegend
Ly6G Alexa Flour 647 (HK1.4)	BioLegend
Ly6G PerCp-Cy5.5 (1A8)	BD Biosciences
CD3e PE (145-2C11)	BioLegend
CD3e PerCP5.5 (17A2)	BioLegend
CD3 Pe-Cy7 (145-2C11)	BioLegend
TCRbeta FITC (H57-597)	BioLegend
CD4 FITC (RM4-4)	Biolegend
CD4 BV785 (GK1.5)	Biolegend
CD8alfa Pacific Blue (53-6.7)	Biolegend
CD8alfa PE (53-6.7)	BioLegend
CD44 AF700 (IM7)	BioLegend
CD25 PE (PC61)	BioLegend
CD25 PeCy7 (PC61)	BioLegend
Foxp3 Pe-Cy5 (FJK-16S)	eBioscience
Foxp3 APC (FJK-16S)	Invitrogen
CD45.1 FITC (A20)	BioLegend
CD45.2 Pe-Cy7 (104)	BioLegend
Neuropilin BV421 (3E12)	BioLegend

Table S2. List of flow cytometry antibodies used.

Antibodies	Clone	Source	dilutions	Catalog #
anti-mCD4-eF450	RM4-5	Invitrogen	1 to 50	48-0042-82
anti-mCD4-PE	RM4-5	Biolegend	1 to 50	100512
anti-mEndomucin Rat	V.7C7	R&D	1 to 200	Sc-65495
anti-mC-kit Goat	polyclonal	R&D	1 to 200	AF1356
anti-mFoxP3 AF488 Rat	FJK.16s	Invitrogen	1 to 50	53-5773-82
anti-mCollagen-IV Rabbit	polyclonal	Abcam	1-300	ab6586
anti-mCD3-biotin Armenian Hamster	145-2C11	Biolegend	1 to 50	100304
Donkey anti-rabbit	AF488	Invitrogen	1 to 200	A-21206
Donkey anti-rabbit AF546	polyclonal	Invitrogen	1 to 200	A10040
Donkey anti-rat AF594	polyclonal	Invitrogen	1 to 200	A-21209
Donkey anti-goat AF680	polyclonal	Invitrogen	1 to 200	A-21084

Reagents		Source	dilutions	Catalog #
DAPI (4’,6-diamidino-2-phenylindole, dihydrochloride)	-	ThermoFisher	1 to 500	62247
RapiClear 1.52	-	SunJin Lab	-	RC152001
Streptavidin BV605	-	Biolegend	1 to 200	405229

Table S3. List of antibodies and reagents used for high-resolution microscopy

Caption for supplementary Videos

Video 1: High-resolution 3D confocal imaging of thick horizontal sections of WT hind paws. c-Kit^{+} HSPCs (red) are nearly absent in WT paws. Paw architecture, visualized via blood vessels, nuclei (DAPI, white), and collagen IV staining (green).

Video 2: c-Kit^{+} HSPCs Accumulate in Inflamed Hind Paw Bone Marrow of CMO Mice. High-resolution 3D confocal imaging of thick horizontal sections of CMO hind paws. c-Kit^{+} HSPCs (red) markedly accumulate in the inflamed paws of CMO mice. HSPCs localize within paw marrow niche, adjacent to sinusoidal vessels labeled with endomucin (blue). The BM architecture, visualized via blood vessels, nuclei (DAPI, white), and collagen IV staining (green), is notably disrupted in inflamed CMO paws.

Video 3: High-resolution 3D confocal imaging of thick horizontal sections of WT hind paws. Low presence of CD3^{+} (blue), CD4^{+} (magenta), FoxP3^{+} (white) regulatory T cells (Tregs) in WT paw marrow.

Video 4: High-resolution 3D confocal imaging of thick horizontal sections of CMO hind paws. CD3^{+} (blue), CD4^{+} (magenta), FoxP3^{+} (white) regulatory T cells (Tregs) accumulate in the marrow of CMO paws. Notably, in CMO mice, Tregs are positioned in close proximity to c-Kit^{+} HSPCs (red), suggesting potential local interactions within the inflamed paw niche.
